# Modelling drift of cold-stunned Kemp's ridley turtles stranding on the Dutch coast

**DOI:** 10.12688/openreseurope.16913.1

**Published:** 2024-02-29

**Authors:** Darshika Manral, Ilse Bos, Mark de Boer, Erik van Sebille

**Affiliations:** 1 Institute for Marine and Atmospheric Research, Utrecht University, Utrecht, 3584 CC, The Netherlands; 2 Department of Biology, Utrecht University, Utrecht, 3584 CC, The Netherlands; 3 Curator Fishes, Reptiles, Amphibians and Invertebrates, Rotterdam Zoo, Rotterdam, 3041 JG, The Netherlands

**Keywords:** Lepidochelys kempii, juvenile sea turtles, critically endangered, cold stunning, Lagrangian modelling, turtles stranding

## Abstract

**Background:**

Every few years juvenile Kemp’s ridley turtles ( *Lepidochelys kempii*) are found stranded on the Dutch coasts. The main population distribution of this critically endangered species primarily inhabits the Gulf of Mexico and east coast of the United States. This study focuses on five reports from the Netherlands over the past 15 years, where juvenile turtles were reported to strand alive during the winter, albeit in a hypothermic state. Between ambient ocean temperatures of 10°C and 13°C, Kemp’s ridley turtles are unable to actively swim and remain afloat on the ocean’s surface, a condition termed *‘cold stunning’*. Understanding their transport in cold-stunned state can help improve the rehabilitation process of stranded turtles.

**Methods:**

Cold-stunned turtles are back-tracked as passive, virtual particles from their stranding location using Lagrangian flow modelling. This study investigates when and where juveniles of the Kemp's ridley turtles cross the threshold temperatures between 10–14° C before stranding by tracking the temperature along the trajectories.

**Results:**

As expected, the simulations show transport of the turtles to the Dutch coast via the English Channel. More surprisingly, the analysis suggests that they likely experience cold-stunning in the southern North Sea region and encounter temperatures below 10°C for only a few days to up to three weeks, and below 12°C for up to a month before stranding.

**Conclusions:**

Adherence to rehabilitation protocols for Kemp’s ridley and monitoring individuals post-release are recommended to improve their long-term survival.

## 1 Introduction

Kemp’s ridley turtles ( *Lepidochelys kempii*) are the smallest and the most endangered of all the sea turtles species ^1^. With 22,341 mature individuals of a single known population and declining numbers over the past three generations, they are critically endangered according to the latest International Union for Conservation of Nature (IUCN) assessment ^2^. The geographic range of Kemp’s ridleys primarily extends from the Gulf of Mexico to the east coast of the United States, and nesting sites are predominantly limited to a narrow stretch of 30 km coastline in the western Gulf of Mexico ^2, 3^. This makes the Kemp’s ridley, along with the flatback sea turtle, the sea turtle species with the most geographically restricted distribution ^4^.

Like other sea turtle species, Kemp’s ridley adults spend most time foraging in shallow coastal waters while offsprings move to the pelagic waters within hours of hatching ^3, 5^. The oceanic stage of young turtles is the least understood stage of most turtle species and is often referred as *‘the lost years’* ^6^. Turtle hatchlings are mainly dispersed by ocean currents and likely feed on planktonic prey in fronts and convergence zones ^5, 7^. Most Kemp’s ridleys recruit as juveniles in coasts along the northern Gulf of Mexico and eastern US coast after spending several months to around two years in the open waters ^3^. Additionally, juvenile turtles between one and two years of age have also been sporadically reported in coasts along the north-east Atlantic Ocean and Mediterranean Sea ^8– 14^. In addition to Kemp’s ridley turtles, the transatlantic transport of young turtles via gyre followed by the North Atlantic Current or Azores Current is also suggested for leatherback ( *Dermochelys coriacea*) and loggerhead ( *Caretta caretta*) turtles ^5, 8, 10, 15, 16^.

In a study comparing trajectories of satellite tagged young turtles and surface drifters, ^17^ suggested active swimming behaviour for young Kemp ridley’s during the oceanic stage. However, since sea turtles are ectotherms, the temperature of the ocean plays a crucial role in different aspects of their life-cycle including growth and activity ^18^. Kemp’s ridley juveniles feeding in the northern Gulf of Mexico and eastern US coast during spring and summer months are known to perform seasonal southward migration in the fall and winter to avoid cold conditions ^19, 20^. Those unable to leave the feeding grounds in time often strand and/or die ^5, 21^. Low temperatures reduce metabolic activity in the turtles, leading to a hypothermic condition termed *‘cold stunning’*. In an experimental study ^22^, observed Kemp’s ridley turtles sluggishly floating at 10-13°C of water temperature. Multiple studies since then have found stranding of cold-stunned Kemp’s ridley turtles at ocean temperature below 10°C ^23– 26^. Further, exposure time to cold conditions ^18^ and wind - speed and direction ^23, 24, 26^, are additional factors identified for occurrence of stranding events.

Strandings of cold-stunned Kemp’s ridley turtles is a yearly phenomenon during the winter in the US east coast ^21, 23, 26^. In the US, juvenile Kemp’s ridleys can start stranding within a day of ocean temperature dropping below 10°C, and these cold conditions can last up to a few weeks, causing an increase in stranded turtles count ^21, 24, 25^. The stranding count can vary from few tens to hundreds of individuals in a year ^21, 26^, with largest stranding of 1,180 Kemp’s ridleys in 2014/2015 at Cape Cod Bay, Massachusetts ^3^. Cold conditions in the north-east Atlantic Ocean can also lead to strandings of juveniles in the UK, Ireland, France, Netherlands and Spain ^8, 10– 13, 27, 28^. These strandings are, however, comparatively sporadic. A recent study by 29 has raised concern that range expansion caused by global warming can result in rise of Kemp’s ridley strandings and, in addition to strandings, Kemp’s ridleys also face threats from human activities like habitat destruction, fisheries, oil spills and illegal harvesting ^1, 3^. Therefore, it is important to continue efforts to protect these endangered and young turtles in the European waters.

Cold-stunned stranded turtles are generally found in poor health conditions and rehabilitation programmes play a crucial role in attempts to save these individuals ^3, 10, 30, 31^, with some individuals known to successfully survive for a few years post-release ^32^. Nevertheless, the long-term survival of rescued individuals is still largely unknown for Kemp’s ridley turtles ^33^. Rehabilitation protocols developed primarily in the US over decades of experience working with stranded turtles, are also referenced in Europe and the UK. However, less rehabilitation experience has been acquired due to small number of strandings and thus, there has been little validation of these protocols for the European strandings. Additionally, these stranded juvenile turtles in the European waters might have a poorer health compared to ones stranded closer to their native habitats in the US, as observed in 28. In order to enhance the survival prospects of endangered and threatened species like Kemp’s ridley sea turtles, it is crucial to have every individual, particularly those nearing reproductive age, released back into the wild to maximize the potential for species preservation ^34^. Even though costly, the translocation of rehabilitated Kemp’s ridley sea turtles contributes to sea turtle welfare and offers additional benefits such as education, research, and collaboration among various organizations involved in the conservation efforts ^33^.

This study focuses on the five strandings of cold-stunned Kemp’s ridley juveniles that occurred in the Netherlands in last 15 years, and investigates their transport in the cold-stunned state. Since this turtle species is not known to forage in the southern North Sea, it is anticipated that they likely face hypothermic conditions along their occasional drift from the North Atlantic Ocean, which could take multiple months. Specifically, the study aims to answer the following questions: *Around which region would Kemp’s ridley turtles have encountered critical threshold water temperatures? How long have they drifted in the cold-stunned state before stranding on the Dutch coast?* These questions can assist in estimating the health status of the stranded individuals (e.g., the last time it might have fed). Although it is not known whether the individuals in the north-east Atlantic Ocean can naturally return to their native grounds as sub-adults or adults ^10^ and contribute to the future populations, the information acquired can improve probability of successful rehabilitation of the stranded individuals of this critically endangered species. Only two of the seven Kemp’s ridley juveniles that have ever been recorded to strand in the Netherlands (five of which in the last 15 years) were successfully rehabilitated to the Gulf of Mexico ^10^.

## 2 Methods

### Dutch strandings

The southern North Sea, bounded on the east by the Dutch coastline, is predominantly a shallow sea (depth less than 100 m). The flow in this region is influenced largely by tides and seasonal winds. The Dutch coastline consists mostly of sand dunes resulting in flat and accessible beaches, with population centres close-by almost everywhere. The study region is further discussed in Section 3. In the past, seven strandings of Kemp’s ridley turtles have been reported in the Netherlands ^11, 12, 35^. Two of those strandings took place in 1954 and 1970, while the others happened in this century with the most recent one in December 2021 ( Table 1). The stranding data was obtained from waarneming.nl, an online platform where different flora and fauna observations in the Netherlands are curated. All of the stranded turtles were alive at the time of reporting, which might reflect the accessibility of Dutch beaches and thus the likelihood that these turtles would not have remained stranded on the beach for long before being found. The Curved Curvature Length (CCL) of these individuals was between 20–30 cm ^, 10– 12, 35^ and were identified as juveniles ^36^.

**Table 1.** Locations and date for stranded juvenile Kemp’s ridley turtles on the Dutch coast used in this study. *Source: waarneming.nl*.

Location	Latitude (°)	Longitude (°)	Date
IJmuiden	52.451733	4.554254	13/01/2007
Westenschouwen	51.7420	3.7578	21/11/2008
Monster	52.0277	4.1591	12/12/2011
Den Helder	52.9627	4.7323	22/12/2014
Westkapelle	51.5242	3.4384	02/12/2021

### Ocean model data and simulation set up

Two-dimensional tracking of particles, backward in time from the stranding locations and dates, are performed using the Parcels Lagrangian framework ^37^. To simulate the ocean conditions in the stranding years, reanalysis data is used which assimilates observations from satellite and in-situ instruments. Daily mean ocean surface currents that include the effect of tides are obtained from the reanalysis data for European North West Shelf along with ocean surface temperatures, all available since 1993 at the spatial resolution of 0.111 *◦ ×* 0.067° ^38^. In addition, the effect of Stokes drift ^39^ on the transport of these particles is accounted for using three-hourly data from 40, available since 1980 at 1.5 km resolution. Since floating turtles might be partially exposed to the atmospheric wind, the effect of wind on their transport is also investigated ^41^. Three-hourly wind at 10 m above sea level is obtained from ERA5 reanalysis data at 0.25° *×* 0.25° resolution ^42^. Of the seven known strandings in the Netherlands, analysis is done for the five strandings (see Table 1) which occurred in years for which ocean surface currents data is available.

In the simulations, 10,000 particles are released at the adjacent coastal cell of each stranding location and traced backward in time ( Table 1) ^43^; an approach also used for tracking marine debris in 44. We assume that the nearshore arrival of the turtles at the stranding location is well approximated using ocean data on currents, tides, waves and wind; although in reality, fine scale coastal processes can be quite complex to simulate. The simulations run backward in time from the reported date of stranding for a total of 120 days, using a fourth-order Runga-Kutta advection algorithm with a time-step of 10 minutes. Particles are advected with a combined flow obtained from summing ocean currents, Stokes drift, and windage as a fraction of the 10 m atmospheric wind. Since it is not known to what extent windage affects the juvenile turtles drift, a sensitivity analysis is done with four settings of windage factor: 0.0% (i.e., no windage), 0.1%, 1.0%, 2.0% and 3.0%. Similar windage settings have been tested for floating marine debris in 45 and 46. The ocean surface temperature is also sampled along the simulated trajectory. Output particle locations and instantaneous temperatures are stored every day in the output file. With an A-grid ocean data and coastal releases, particles tend to get stuck on coastlines relatively easily ^37^. To avoid particles from getting stuck, an anti-beaching kernel is applied, where if a particles enters a land grid cell, it is pushed back into the water with an arbitrary velocity of 1 m/s resulting in 600 m displacement (with a 10 minutes time step) perpendicular to the shoreline ^44^. However, particles can still get stuck in regions very close to convoluted coastlines or on small islands; these particles are then marked as beached.

### Defining cold-stunning event

Literature suggests that Kemp’s ridley turtles can start to show signs of cold stunning at ocean surface below 13°C ^22^ and are known to beach in a cold-stunned state when ocean surface temperatures drop below 10°C ^23– 25^. Cold ocean temperatures impair the ability of sea turtles to actively swim, making them passively float at the surface ^22^ and drift with the ocean currents in this cold-stunned state. Therefore, the movement of the cold-stunned Kemp’s ridley turtles is simulated in this study as passively drifting particles at the ocean’s surface (also suggested in 23 and 26). The most recent event before stranding when the interpolated ocean surface temperature on a particle trajectory drops below a threshold temperature is extracted from its trajectory and marked as a cold-stunning event, similar to the approach used in 26. We assume that turtles can swim above the threshold temperature and hence, their movement cannot be simulated with passive drift. In nature, this switch from active to passive drift is indeed gradual and depends on the health status of the individual turtle; therefore, we examine three threshold temperatures ( *T _c_* : 10°C, 12°C and 14°C) in this analysis ^43^.

## 3 Results

**Figure 1.**
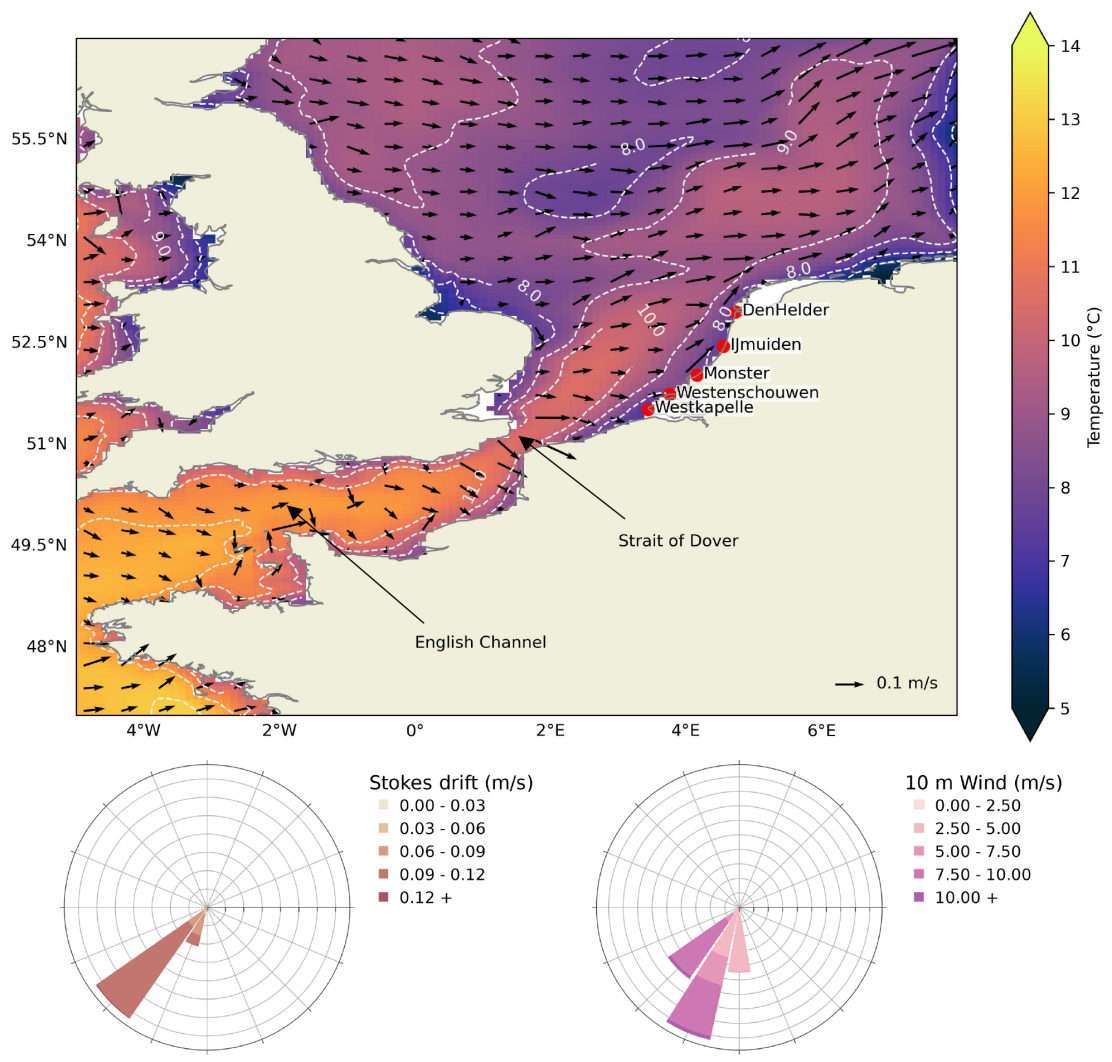
Study region of European North West Shelf. *top:* Mean surface currents *(arrows)* and temperature *(colormap)* for the December months of years 2006, 2008, 2011, 2014 and 2021 ^38^ close to the stranding dates of Kemp’s ridley turtles in the Netherlands and stranding locations are also marked with *red dots* ( Table 1). The *dashed* lines show the temperature contour. *bottom:* Wind-rose diagrams show the mean Stokes drift *(left)* and 10 m wind *(right)* for the same months and for the region shown here. Land and coastlines are obtained from python package *cartopy*.

In winter, average ocean temperature in the southern North Sea can reach below 10°C ( Figure 1), creating conditions for cold stunning and potential strandings of juvenile Kemp’s ridley turtles present in this region. Due to sea surface temperature of 8–9°C to the east of the United Kingdom, it is unlikely that the turtles could have survived the transport from the north. Relatively higher temperatures are observed south of the Strait of Dover due to the transport of subtropical heat via the Gulf Stream and North Atlantic Current. In particular, the lowest temperatures in the southern North Sea are observed along the coastlines and the effect of influx of warmer water from the North Atlantic Current is visible away from the coasts. In addition, ocean currents, Stokes drift and wind are predominantly south-westerly in December ( Figure 1). As can be seen in the animations ^47^, these environmental factors result in transport of passive particles via the English Channel and through the Strait of Dover, before stranding on the Dutch coast. However, since turtles that are not cold-stunned are active swimmers, we focus on their transport once the ocean surface temperatures along the trajectories drop below selected critical thresholds.

### Simulations without windage

**Figure 2.**
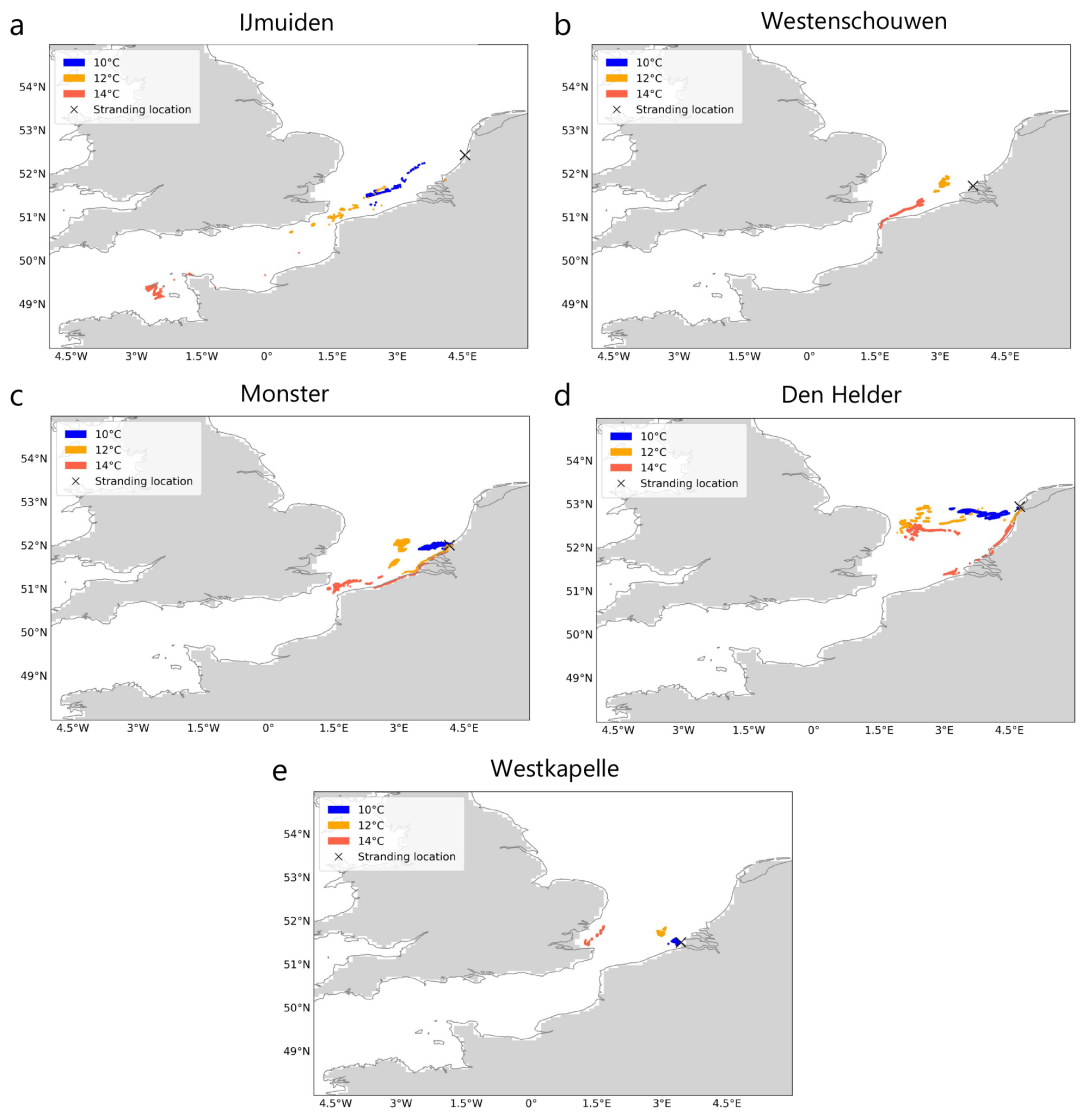
Locations where threshold temperatures were crossed before stranding. For five recent Kemp’s ridley strandings in the Netherlands ( **A**– **E**), locations where threshold ocean surface temperatures ( *T _c_* : 10°, 12° and 14° C) were crossed by particles during Lagrangian simulations with currents and Stokes drift only (i.e., no windage) are shown. Stranding locations are marked for reference. Land mask is obtained from the ocean model ^38^ and coastlines from python package *cartopy*.

**Figure 3.**
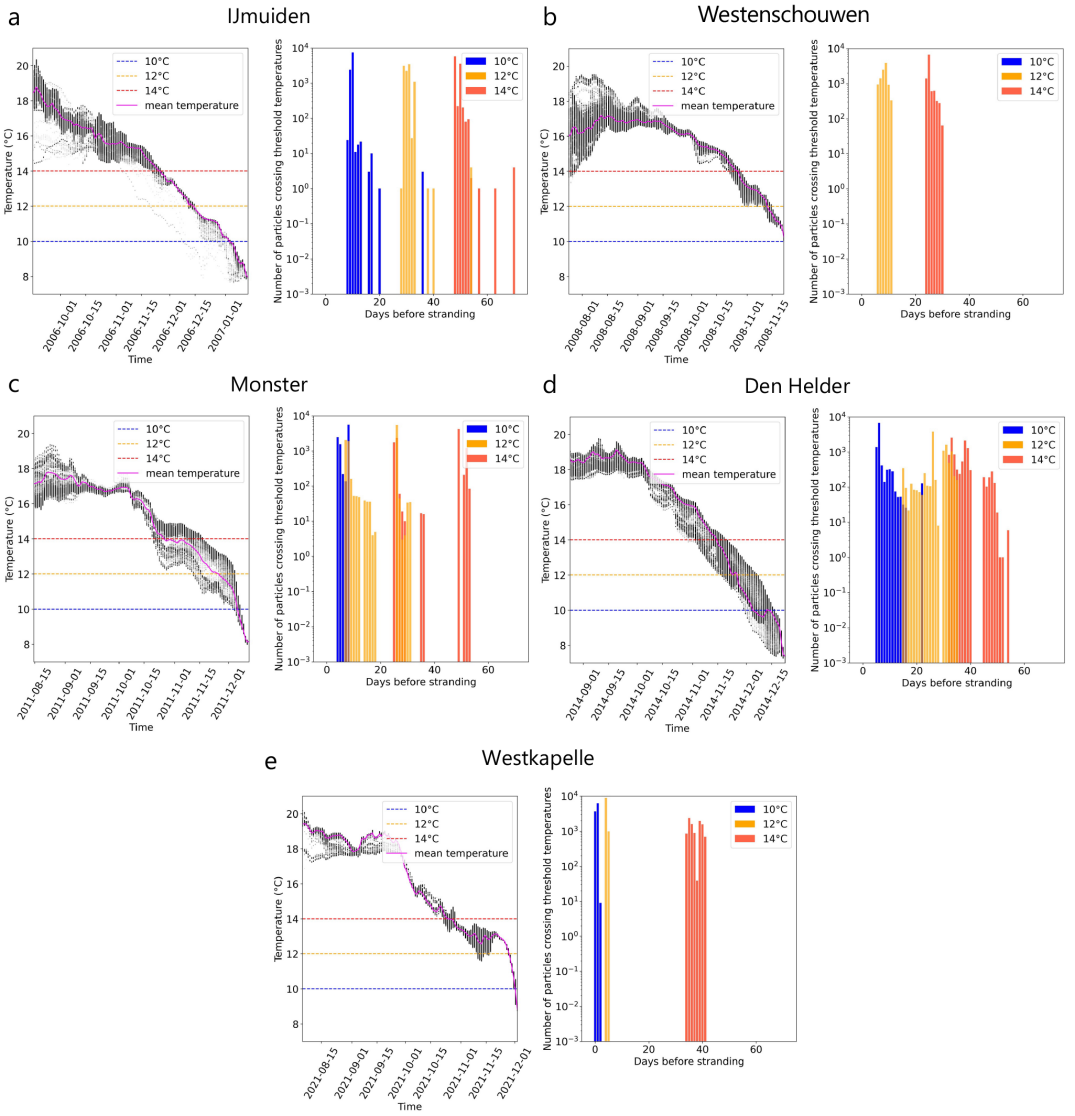
Days before stranding when threshold temperatures were crossed. For five recent Kemp’s ridley strandings in the Netherlands ( **A**– **E**), *(left)* the temperature distribution of all drifting parti- cles *(black dots)* and temperature mean *(pink line)* over time before arriving at the stranding location using Lagrangian simulations with currents and Stokes drift only (i.e., no windage) are shown. Critical threshold temperatures analysed in this study ( *T _c_* : 10°, 12° and 14° C) are marked for reference *(dashed line)*. *(right)* Number of particles crossing threshold temperatures for the last time before stranding.

In the simulations due to currents and Stokes drift only (i.e., with a windage of 0.0%), particle trajectories always drop below threshold temperatures ( *T _c_* ) of 10°, 12°and 14°C within the southern North Sea region ( Figure 2) with the exception of the stranding at IJmuiden, where particles experienced temperatures below 14°C even before crossing the English Channel ( Figure 2a). As expected, the distance from the stranding location decreases as the lower thresholds are crossed ( Figure 2). The continuous - and in the case of the strandings at Monster and Westkapelle steep - decrease in ocean surface temperature along the trajectories as particles approach the stranding location can also be observed in the temperature time series ( Figure 3a–e *(left)*).

In most cases, stranding occurred once *T _c_* = 10°C threshold was crossed by particles. For IJmuiden, Monster and Den Helder, temperatures of approximately 8°C were experienced by particles a few days before strandings ( Figure 3a,c,d). However, for the stranding at Westenschouwen, the ocean surface temperature along the trajectories never dropped below *T _c_* = 10°C ( Figure 3b *(left)*). Most particles crossed the lowest *T _c_* = 10°C a few days (Monster and Westkapelle) to almost two weeks (IJmuiden and Den Helder) before stranding ( Figure 3a,c–e *(right)*). Particles crossed *T _c_* = 12°C approximately a few days to a month before stranding. Finally, particles crossed *T _c_* = 14°C three to six weeks before stranding.

### Simulations with windage

**Figure 4.**
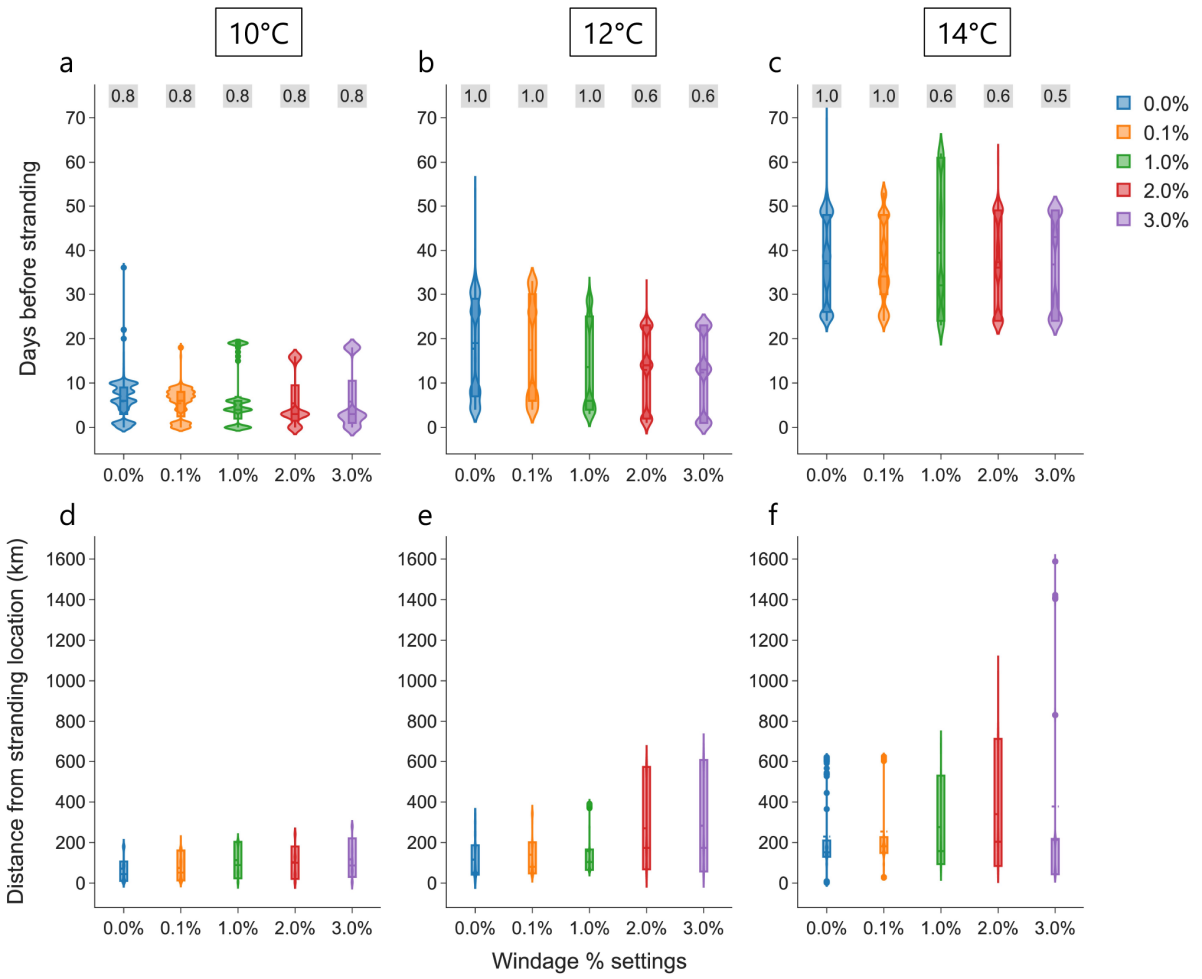
Effect of windage settings. Violin plots of days before stranding ( **a**– **c**) and distance of each particle from the respective stranding locations ( **e**– **f**) when a threshold temperature is crossed under the influence of currents, Stokes drift and different windage settings (0.0%, 0.1%, 1.0%, 2.0% and 3.0%) considered in the analysis is shown. The text in *grey* box on top represents the proportion of total number of particles ( *n* = 50,000) crossing the threshold at a windage setting.

Simulations from all the stranding locations are combined to investigate the additional effect of four different percentages of 10 m atmospheric wind on the transport of particles: 0.1%, 1.0%, 2.0% and 3.0%. For each windage setting, the time and distance between cold-stunning and stranding increases with *T _c_* ; a similar trend to the case without windage ( Figure 4). For the *T _c_* = 10°C threshold, there is a marginal decrease in the observed mean of time between cold-stunning and stranding, and an increase in the distance from the stranding location with increasing windage ( Figure 4a,d). In the Westenschouwen stranding, particles did not cross the *T _c_* = 10°C threshold for any of the windage settings (see Figure3b *(left)* and 47). In addition to Figure 4, a summary of windage effect on cold stunning event for each stranding locations is provided in the Supplementary Information.

At the higher *T _c_* = 12°C and *T _c_* = 14°C thresholds, the time between cold-stunning and stranding show a similar decrease in mean for increasing windage as *T _c_* = 10°C. The distance from the stranding location increased considerably for higher windage of 2.0% and 3.0%. Note that the fraction of particles that crossed a certain *T _c_* decreased with high windage at higher *T _c_* (see Figure 4). This can be primarily attributed to increasing number of beached particles in the complex coastal regions and islands in the simulations: ~2–4% and approximately 50% of *n* = 50, 000 particles beached at low and high windage, respectively. High windage (in addition to currents and Stokes drift) can transport particles further into the land which cannot be successfully displaced to the sea by the anti-beaching kernel. In case of the strandings at Monster and Westkapelle with 2.0–3.0% windage, particles originating from the UK coast with low ocean temperatures arrived at the stranding locations within a few days, without encountering temperatures above 10°C ^47^.

## 4 Discussion

In all the seven historical strandings on the Dutch coast, juvenile Kemp’s ridley turtles were found alive at the time of reporting. However, only two turtles could be successfully rehabilitated to their native habitat in Gulf of Mexico raising questions about the shortcomings in rehabilitation protocols or their adequate implementation. Using Lagrangian simulations with surface currents and Stokes drift, five recent Kemp’s ridley strandings analysed in this study show that turtles might have continuously encountered ocean surface temperatures below 14°C from locations most likely in the southern North Sea and up to the English Channel ( Figure 2). As previously noted in 10, these simulations further support the notion of Kemp’s ridley juvenile turtles reaching the North Sea via the English Channel. For the stranding at Westenschouwen in November 2008, it was observed that the juvenile Kemp’s ridley turtle might not have faced temperatures below 10°C indicating that temperatures between 10–12°C can also result in stranding. Differences in physiological conditions can also be responsible in this case but its analysis is beyond the scope of this study.

The decreasing distance from stranding location and temperature as particles approached the stranding location ( Figure 2 and Figure 3) indicates that if the turtles start developing symptoms of cold stunning at 14°C, they are likely to further face lower temperatures which could deteriorate their condition even more. Hence, turtles are unable to escape from the southern North Sea region to the relatively warmer Atlantic waters ( Figure 1). While an increase in windage values did result in increase in distance from the stranding location, it did not have much effect on the time between cold-stunning and stranding ( Figure 4). This timescale between crossing the threshold temperatures and the stranding on the Dutch coast varied in different stranding events from (i) a day to three weeks for *T _c_* = 10°C, (ii) a few days to a month for *T _c_* = 12°C, and (iii) three weeks to two months for *T _c_* = 14°C. Low metabolism in cold temperatures might be the reason turtles survive this transport over several days, however, it is unclear how long juvenile turtles can endure these conditions.

The possible timescales of drifting in cold-stunned state for few days to two to three weeks (below 10°C) might indicate similar physiological conditions to those in multiple strandings reported within a day to up to a few weeks of temperatures between 8°and 11°C in the east US coast, for e.g. as observed in 24 and 25. This indicates similar protocol requirements for rehabilitation of Kemp’s ridley turtles in the Netherlands. Therefore, it is recommended that rehabilitation protocols are adequately implemented and individuals are further tracked upon release to assess long-term impact of rehabilitation ^32, 33, 48^. For future strandings, the approach used in this study can even be applied to obtain real-time insights on the individual’s history in the cold-stunned state and accordingly take necessary steps for rehabilitation. Additionally, these rehabilitation opportunities should be used to raise awareness about threats faced by wild species and, educate and engage local public towards collaborative conservation efforts ^33^.

Even though, Kemp’s ridley juveniles are known to forage in the north-east Atlantic Ocean ^28, 48^, their transport into the North Sea is considered as an occasional drift ^10^. Additional research could investigate the seasonal food availability in the southern North Sea to assess if these turtles were possibly feeding in this region prior to the onset of winter. Furthermore, it remains to be investigated if conclusions drawn from this study also apply to juvenile Kemp’s ridley strandings on other western European coasts.

## Ethics and consent

Ethical approval and consent were not required.

## Data Availability

The Copernicus Marine Service ocean data used in this study can be downloaded from https://doi.org/10.48670/moi-00059 ^38^ and https://doi.org/10.48670/moi-00060 ^40^. The ERA 5 wind data can be downloaded from https://doi.org/10.24381/cds.adbb2d47 ^42^. Data publication platform of Utrecht University: kemps_ridleys_dutch_strandings. https://doi.org/10.24416/UU01-6NPX5Y ^47^. This project contains the following underlying data: File **simulation_output_files.zip**:Simulation outputs for different windage setting analysed in this study. Windage settings: 0.0%, 0.1%, 1.0%, 2.0% and 3.0% are abbreviated as 0pWind, 01pWind, 1pWind, 2pWind and 3pWind, respectively. Same structure is used in the folders below. Folder **animations/xpWind**: Animations of simulations are available in this folder within subfolders of each windage setting. Folders **analysis/xpWind**: Analysis of cold stunning event for each particle per simulation are available in subfolders for each windage setting. Folder **analysis/windage_effect_per_station**: Effect of each windage setting on the extracted cold stunning event is shown for each station. Data are available under the terms of the Creative Commons Attribution 4.0 International license (CC-BY 4.0). Analysis code available from: https://github.com/OceanParcels/Kempsridley_turtle_strandings Archived analysis code at time of publication: https://zenodo.org/doi/10.5281/zenodo.10450425 ^43^ License: Data are available under the terms of the Creative Commons Attribution 4.0 International license (CC-BY 4.0).
