# Modelling drift of cold-stunned Kemp's ridley turtles stranding on the Dutch coast

**DOI:** 10.12688/openreseurope.16913.3

**Published:** 2024-10-25

**Authors:** Darshika Manral, Ilse Bos, Mark de Boer, Erik van Sebille

**Affiliations:** 1 Institute for Marine and Atmospheric Research, Utrecht University, Utrecht, 3584 CC, The Netherlands; 2 Department of Biology, Utrecht University, Utrecht, 3584 CC, The Netherlands; 3 Curator Fishes, Reptiles, Amphibians and Invertebrates, Rotterdam Zoo, Rotterdam, 3041 JG, The Netherlands

**Keywords:** Lepidochelys kempii, juvenile sea turtles, critically endangered, cold stunning, Lagrangian modelling, turtles stranding

## Abstract

**Background:**

Every few years, juvenile Kemp’s ridley turtles ( *Lepidochelys kempii*) are stranded on the Dutch coasts. The main population distribution of this critically endangered species primarily inhabits the Gulf of Mexico and the east coast of the United States. This study focuses on five reports from the Netherlands between 2007 and 2022, where juvenile turtles were reported to strand alive during the winter, albeit in a hypothermic state. At ambient ocean temperatures between 10°C and 13°C, Kemp’s ridley turtles begin to show an inability to actively swim and remain afloat on the ocean’s surface, a condition termed *‘cold stunning’*. Understanding their transport in cold-stunned state can help improve the rehabilitation process of stranded turtles.

**Methods:**

Cold-stunned turtles are back-tracked as passive, virtual particles from their stranding location using Lagrangian flow modelling. This study investigates when and where these juvenile turtles cross the threshold temperatures between 10° C and 14° C before stranding by tracking the temperature along the trajectories.

**Results:**

As expected, the simulations show the transport of the cold-stunned turtles via the English Channel. More surprisingly, the analysis suggests they likely experience cold-stunning in the southern North Sea region and encounter temperatures below 10°C for only a few days to up to three weeks, and below 12°C for up to a month before stranding.

**Conclusions:**

The estimate of cold-stunned drift duration of the turtles provides additional knowledge about their health status at the time of stranding. Adherence to rehabilitation protocols for Kemp’s ridley and post-release monitoring are recommended to improve their long-term survival.

## Introduction

1 Kemp’s ridley turtles ( *Lepidochelys kempii*) are the smallest and the most endangered sea turtle species ^1^. With 22,341 mature individuals of a single known population and declining numbers over the past three generations, they are critically endangered according to the latest International Union for Conservation of Nature (IUCN) assessment in 2019 ^2^. The geographic range of Kemp’s ridleys primarily extends from the Gulf of Mexico to the east coast of the United States, and nesting sites are predominantly limited to a narrow stretch of 30 km coastline in the western Gulf of Mexico ^2, 3^. This makes the Kemp’s ridley, along with the flatback sea turtle, the sea turtle species with the most geographically restricted distribution ^4^.

Like other sea turtle species, Kemp’s ridley adults spend most time foraging in shallow coastal waters while offsprings move to the pelagic waters within hours of hatching ^3, 5^. The oceanic stage of young turtles is the least understood stage of most turtle species and is often referred to as *‘the lost years’* ^6^. Turtle hatchlings are mainly dispersed by ocean currents and likely feed on planktonic prey in fronts and convergence zones ^5, 7^. Most Kemp’s ridleys recruit as juveniles on coasts along the northern Gulf of Mexico and eastern US coast after spending several months to around two years in the open waters ^3^. Additionally, juvenile turtles between one and two years of age have also been sporadically reported on coasts along the north-east Atlantic Ocean and Mediterranean Sea ^8– 14^. In addition to Kemp’s ridley turtles, the transatlantic transport of young turtles via gyre followed by the North Atlantic Current or Azores Current is also suggested for leatherback ( *Dermochelys coriacea*) and loggerhead ( *Caretta caretta*) turtles ^5, 8, 10, 15, 16^.

In a study comparing trajectories of satellite-tagged young turtles and surface drifters, 17 suggested active swimming behaviour for young Kemp’s ridleys during the oceanic stage. However, since sea turtles are ectotherms, the ocean’s temperature plays a crucial role in different aspects of their life cycle including growth and activity ^18^. Kemp’s ridley juveniles feeding in the northern Gulf of Mexico and the eastern US coast during spring and summer months are known to perform seasonal southward migration in the fall and winter to avoid cold conditions ^19, 20^. Those unable to leave the feeding grounds in time often strand and/or die ^5, 21^. Low temperatures reduce metabolic activity in the turtles, leading to a hypothermic condition termed *‘cold stunning’*. In an experimental study ^22^, Kemp’s ridley turtles started sluggishly floating between water temperatures of 10°C and 13°C. Multiple studies since then have observed strandings of alive or dead cold-stunned Kemp’s ridley turtles at ocean temperature below 10°C ^23– 26^. 18 suggested that the duration of exposure to cold conditions is a factor in the survivability of the turtles. Furthermore, due to the effect of flotation, local surface currents, wind and waves, also influence when and where the turtles strand ^23, 24, 26^.

Strandings of cold-stunned Kemp’s ridley turtles are a yearly phenomenon during the winter along the US east coast ^21, 23, 26^. In the US, juvenile Kemp’s ridleys can start stranding within a day of ocean temperature dropping below 10°C, and these cold conditions can last up to a few weeks, causing an increase in stranded turtles count ^21, 24, 25^. The stranding count can vary from a few tens to hundreds of individuals in a year ^21, 26^, with the largest stranding of 1,180 Kemp’s ridleys in 2014/2015 at Cape Cod Bay, Massachusetts ^3^. Cold conditions in the north-east Atlantic Ocean can also lead to strandings of juveniles in the UK, Ireland, France, Netherlands, and Spain ^8, 10– 13, 27, 28^. These strandings are, however, comparatively sporadic. A recent study by 29 has raised concern that range expansion caused by global warming can result in the rise of Kemp’s ridley strandings. In addition to strandings, Kemp’s ridleys also face threats from human activities like habitat destruction, fisheries, oil spills, and illegal harvesting ^1, 3^. Therefore, it is important to continue efforts to protect these young and endangered turtles and to expand on our understanding of their strandings along the European coastlines.

Cold-stunned stranded turtles are generally found in poor health conditions and rehabilitation programmes play a crucial role in attempts to save these individuals ^3, 10, 30, 31^, with some individuals known to successfully survive for a few years post-release ^32^. Nevertheless, the long-term survival of rescued individuals is still largely unknown for Kemp’s ridley turtles ^33^. Rehabilitation protocols developed primarily in the US over decades of experience working with stranded turtles, are also referenced in Europe and the UK. However, less rehabilitation experience has been acquired due to a small number of strandings and thus, there has been little validation of these protocols for the European strandings. Additionally, these stranded juvenile turtles in the European waters might have poorer health compared to ones stranded closer to their native habitats in the US, as observed in 28. Rehabilitating and releasing endangered or threatened species like Kemp’s ridley sea turtles into the wild may enhance their survival prospects and contribute to species conservation ^34^, especially in the case of individuals nearing reproductive age. Even though costly, the translocation of rehabilitated Kemp’s ridley sea turtles contributes to sea turtle welfare and offers additional benefits such as education, research, and collaboration among various organizations involved in the conservation efforts ^33^.

This study focuses on the five strandings of cold-stunned Kemp’s ridley juveniles that occurred in the Netherlands between 2007 and 2022 and investigates their transport in the cold-stunned state. Since this turtle species is not known to forage in the southern North Sea, it is anticipated that they likely face hypothermic conditions along their occasional drift from the North Atlantic Ocean, which could take multiple months. Specifically, using Lagrangian ocean modelling, the study aims to answer the following questions: *Around which region would Kemp’s ridley turtles have encountered critical threshold water temperatures? How long have they drifted in the cold-stunned state before stranding on the Dutch coast?* These questions can assist in estimating the health status of the stranded individuals (e.g., the last time it might have fed).

Previously, pathways of cold-stunned or dead turtles have been studied using different methods like tracking of drift bottles experiment and satellite-tracked drifters released offshore, and forward and backward in time virtual particle simulations using ocean data ^26, 35, 36^. In addition, these studies then analyse environmental factors like temperature, wind, and currents data to interpret the stranding patterns of cold-stunned turtles. Here, we combine these approaches to model the drift of cold-stunned turtles from their observed stranding locations and dates using surface currents, waves, and wind data, along with temperature sampling along the trajectory of virtual particles in a previously unstudied region.

The strandings of juvenile Kemp’s ridley turtles in the Netherlands are unique, though rare, as they always stranded alive, unlike loggerhead, green, and leatherback turtles, which arrive at different life stages and are often dead or in various stages of decomposition ^10^. This higher survival rate may be attributed to the observed higher tolerance of Kemp’s ridleys to lethal temperatures of 5–6.5°C for 20–24 hours compared to loggerhead or green turtles ^22^. Secondly, Kemp’s ridleys stranding in the Netherlands belong to a single population originating from the Gulf of Mexico ^2^, while species such as loggerhead turtles, which have multiple subpopulations in the Atlantic Ocean ^37^, can arrive in the Netherlands from different regions. This conclusion is based on an analysis of loggerhead turtles that stranded in the Netherlands in 2023 and 2024, conducted by the *Gendika B.V.* laboratory in Veendam (Netherlands), using the method described in 38 (unpublished). Although it is not known whether the young Kemp’s ridley individuals in the north-east Atlantic Ocean can naturally return to their native grounds as sub-adults or adults ^10^ and contribute to the future populations, the information acquired can improve the probability of successful rehabilitation of the stranded individuals of this critically endangered species. Only two of the seven Kemp’s ridley juveniles recorded to strand in the Netherlands until 2022 survived and were successfully rehabilitated to the Gulf of Mexico ^10^.

## 2 Methods

### Dutch strandings

**Figure 1.**
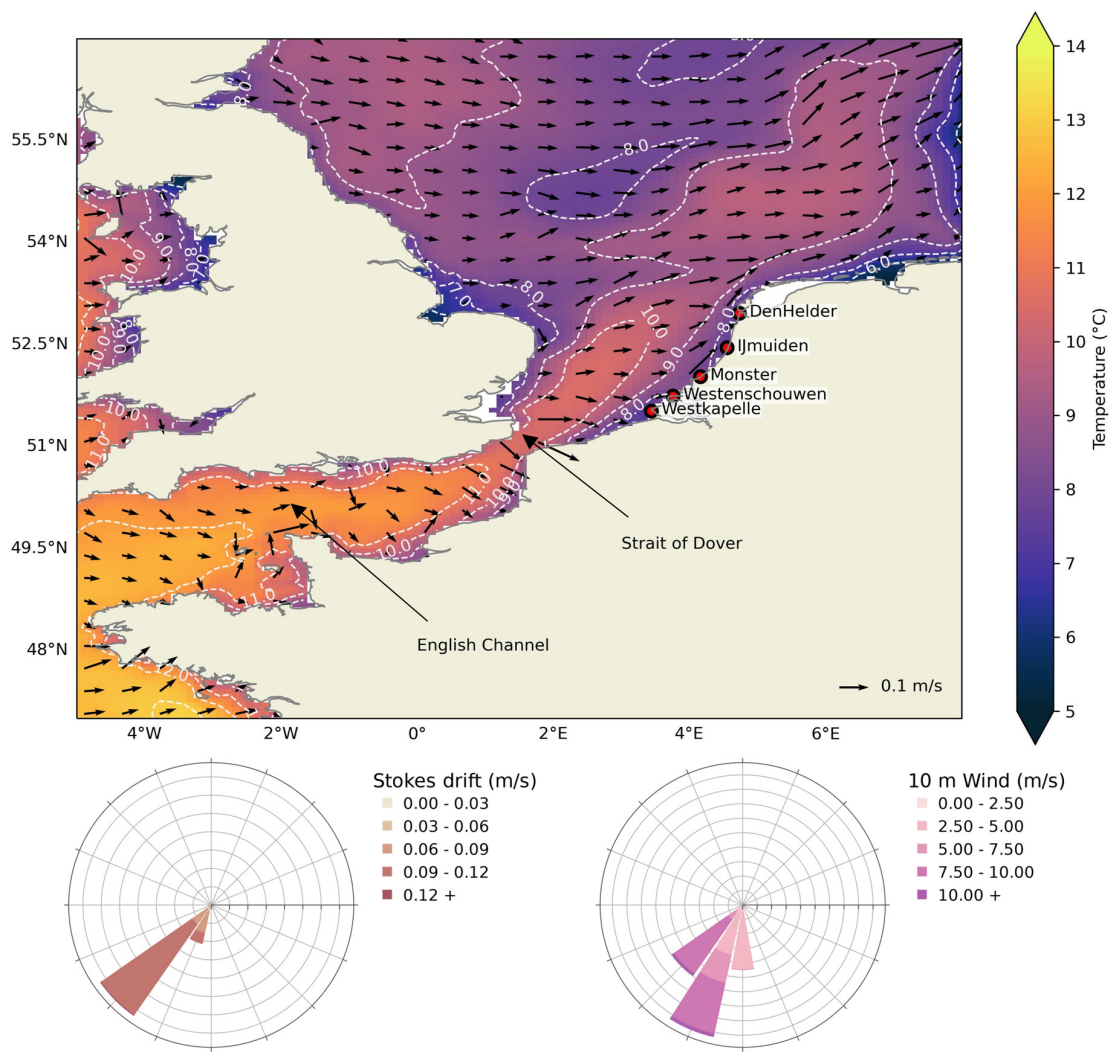
Study region of the European Northwest Shelf. *top:* Mean surface currents *(arrows)* and temperature *(colormap)* for the December months of years 2006, 2008, 2011, 2014, and 2021 ^44^ close to the stranding dates of Kemp’s ridley turtles in the Netherlands, and stranding locations are also marked with *red circles* ( Table 1). The *dashed* lines show the temperature contour. *bottom:* Wind-rose diagrams show the mean Stokes drift *(left)* and 10 m wind *(right)* for the same months and for the region shown here. Land and coastlines are obtained from python package *cartopy* ^61^.

Seven strandings of Kemp’s ridley turtles have been reported in the Netherlands until 2022 ^11, 12, 39^. Two of those strandings took place in December of 1954 and 1970, while the others happened in this century ( Table 1). The 2007 stranding location was obtained from 40 and the rest from waarneming.nl, a citizen-science-based online platform where different flora and fauna observations in the Netherlands are curated. These strandings were also confirmed in the literature, with details about turtles’ status ^10– 12^. All the stranded turtles were alive at the time of reporting, which might reflect the accessibility of flat and sandy Dutch beaches from nearby population centres, increasing the likelihood that these turtles were not stranded for long before being found. The curved carapace length notch to tip (CCLn-t) (see Figure 1b in 41) of these individuals was between 20–30 cm ^10– 12, 39^ and were identified as juveniles ^42^.

**Table 1.** Locations and date for stranded juvenile Kemp’s ridley turtles on the Dutch coast used in this study. *Source:* 2007 stranding from 40 and the rest from *waarneming.nl*.

Location	Latitude (°)	Longitude (°)	Date
IJmuiden	52.451733	4.554254	13/01/2007
Westenschouwen	51.7420	3.7578	21/11/2008
Monster	52.0277	4.1591	12/12/2011
Den Helder	52.9627	4.7323	22/12/2014
Westkapelle	51.5242	3.4384	02/12/2021

### Ocean model data and simulation set up

Two-dimensional tracking of particles, backward in time from the stranding locations and dates, is performed using the Parcels Lagrangian framework ^43^. To simulate the ocean conditions in the stranding years, reanalysis data is used which assimilates observations from satellite and in-situ instruments. Daily mean ocean surface currents that include the effect of tides are obtained from the reanalysis data for the European North West Shelf along with ocean surface temperatures, all available since 1993 at the spatial resolution of 0.111° × 0.067° (~ 7 km) ^44^. In addition, the effect of Stokes drift (a net transport in the direction of wave propagation due to surface waves) ^45^ on the transport of these particles is accounted for using three-hourly data from 46, available since 1980 at ~1.5 km resolution (0.0135° × 0.0303°). Since floating turtles might be partially exposed to the atmospheric wind, the direct effect of wind on their transport is also investigated ^47^. Three-hourly wind at 10 m above sea level is obtained from ERA5 reanalysis data at 0.25° *×* 0.25° resolution (~17.51 km × 27.83 km, computed at 51° N) ^48^. Of the seven known strandings in the Netherlands until 2022, analysis is done for the five strandings since 2007 (see Table 1) for which ocean surface currents data is available.

In the simulations, 10,000 particles are released at the adjacent coastal cell of each stranding location and traced backward in time ( Table 1) ^49^; an approach also used for tracking marine debris in 50. We assume that the nearshore arrival of the turtles at the stranding location is well approximated using ocean data on currents, tides, waves, and wind; although in reality, fine-scale coastal processes can be quite complex to simulate. The simulations run backward in time from the reported date of stranding for a total of 120 days, using a fourth-order Runga-Kutta advection algorithm with a time-step of 10 minutes. Particles are advected with a combined flow obtained from summing ocean currents (with tides), Stokes drift, and windage as a fraction (i.e., windage factor) of the 10 m atmospheric wind, similar to the formulation used for modeling drift of floating marine debris in 51 and 52.

The combination of Stokes drift and windage combined defines the ‘leeway’ drift of a floating object relative to the ambient current ^47, 53^. However, since wave models that compute Stokes drift are not always available, and Stokes drift is indirectly driven by local wind, just like windage, the resulting leeway is for simplicity often estimated from the 10 m wind ^47, 54^. Estimates of leeway for floating sea turtle carcasses range from 1 to 3.6% of 10 m wind ^55, 56^, similar to that of medium-sized marine debris ^57, 58^. Furthermore, in an experiment and modeling based study by 59, it was shown that windage accounts for about one-third of the leeway for a half submerged spherical buoy (diameter = 35 cm), which is comparable to the length of juvenile Kemp’s ridley turtles. This suggests that windage contribution likely varies between 0.3 and 1.2% for these turtles.

Given the uncertainties of leeway contribution to the drift of the alive juvenile turtles, we perform a sensitivity analysis with five settings of windage factor: 0.0% (i.e., no windage), 0.1%, 1.0%, 2.0%, and 3.0%. The ocean surface temperature is also sampled along the simulated trajectory. Output particle locations and instantaneous temperatures are stored daily in the output file. With an A-grid ocean data and coastal releases, particles can get stuck on coastlines relatively easily ^43^. To avoid particles from getting stuck, an anti-beaching kernel is applied, where if a particle enters a land grid cell, it is pushed back into the water with an arbitrary velocity of 1 m/s resulting in 600 m displacement (with a 10 minute time step) perpendicular to the shoreline ^50^. However, particles can still get stuck in regions very close to convoluted coastlines or on small islands; these particles are then marked as beached.

### Identifying the cold-stunning event

Due to the flotation of the cold-stunned Kemp’s ridley turtles, their movement is simulated as passively drifting virtual particles at the ocean’s surface (also suggested in 23 and 26). The most recent event before stranding when the interpolated ocean surface temperature on a particle trajectory drops below a threshold temperature is extracted from its trajectory and marked as a cold-stunning event, similar to the approach used in 26. We assume that turtles can swim above the threshold temperature and hence, their movement cannot be simulated with passive drift. In nature, this switch from active to passive drift is indeed gradual and depends on the health status of the individual turtle; therefore, we examine three threshold temperatures ( *T _c_* : 10°C, 12°C and 14°C) in this analysis. Our approach differs from other studies where a single temperature (10.5°C) was selected to consider turtles as cold-stunned ^26^, or where it was assumed that young turtles in the first year of their drift die if the temperature drops below 10°C ^36^.

## 3 Results

The southern North Sea, bounded on the east by the Dutch coastline, is predominantly a shallow sea (depth less than 100 m). The flow in this region is influenced largely by tides and seasonal winds. In winter, the average ocean temperature in the southern North Sea can reach below 10°C ( Figure 1), creating conditions for cold stunning and potential strandings of juvenile Kemp’s ridley turtles present in this region. Due to the sea surface temperature of 8–9°C to the east of the United Kingdom, it is unlikely that the turtles could have survived the transport from the north. Relatively higher temperatures are observed south of the Strait of Dover due to the transport of subtropical heat via the Gulf Stream and North Atlantic Current. In particular, the lowest temperatures in the southern North Sea are observed along the coastlines and the effect of influx of warmer water from the North Atlantic Current is visible away from the coasts. In addition, ocean currents, Stokes drift, and wind are predominantly south-westerly in December ( Figure 1). As can be seen in the animations ^60^, these environmental factors result in the transport of passive particles via the English Channel and through the Strait of Dover, before stranding on the Dutch coast. However, since turtles that are not cold-stunned are active swimmers, we focus on their transport once the ocean surface temperatures along the trajectories drop below selected critical thresholds.

### Simulations without windage

**Figure 2.**
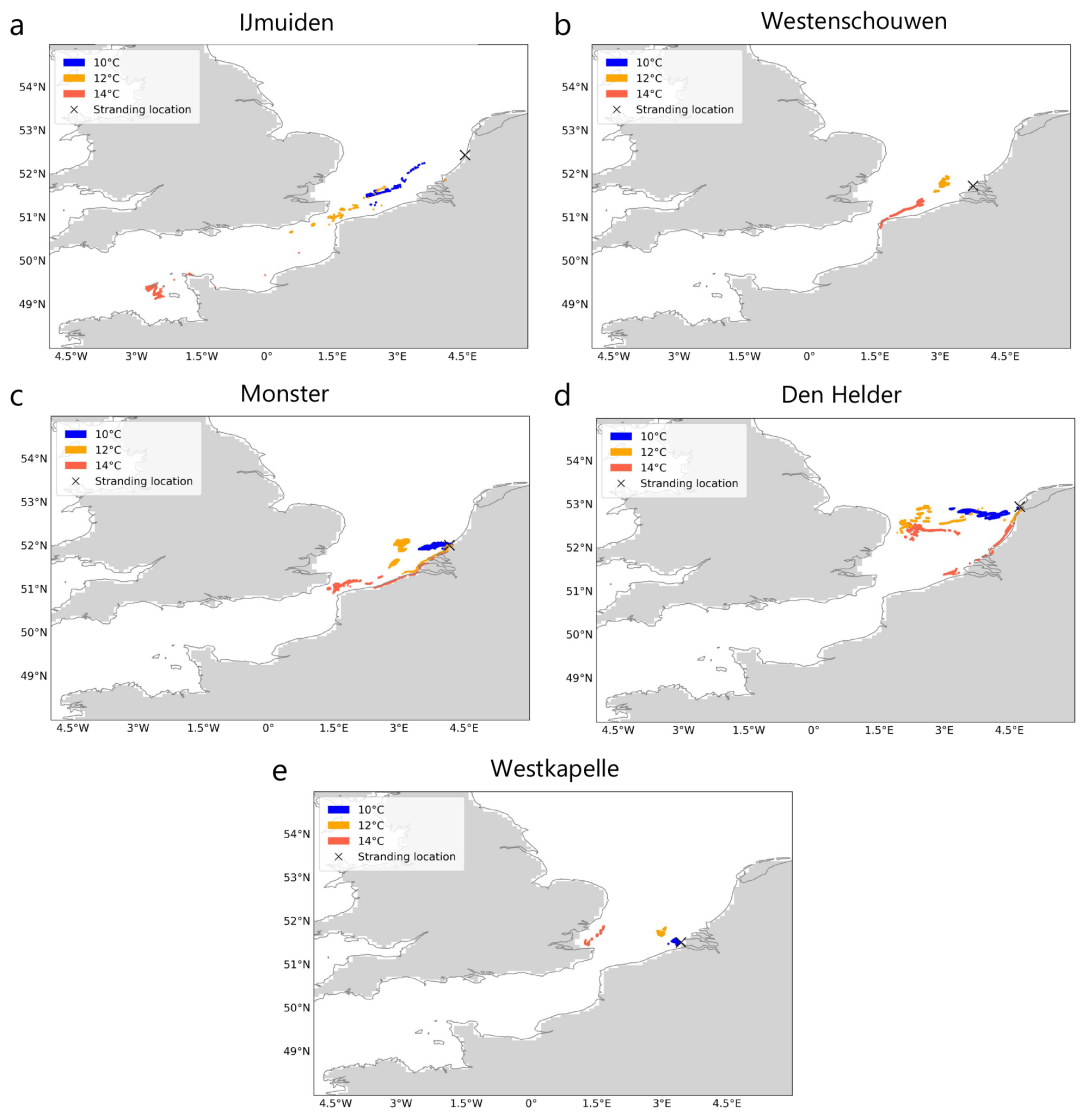
Locations where threshold temperatures were crossed before stranding. For five recent Kemp’s ridley strandings in the Netherlands between 2007 and 2022 ( **A**– **E**), locations where threshold ocean surface temperatures ( *T _c_* : 10°, 12° and 14° C) were crossed by particles during Lagrangian simulations with currents and Stokes drift only (i.e., no windage) are shown. Stranding locations are marked for reference. The land mask is obtained from the ocean model ^44^ and coastlines from python package *cartopy* ^61^. The animations of the back-tracked particles are provided in 60.

**Figure 3.**
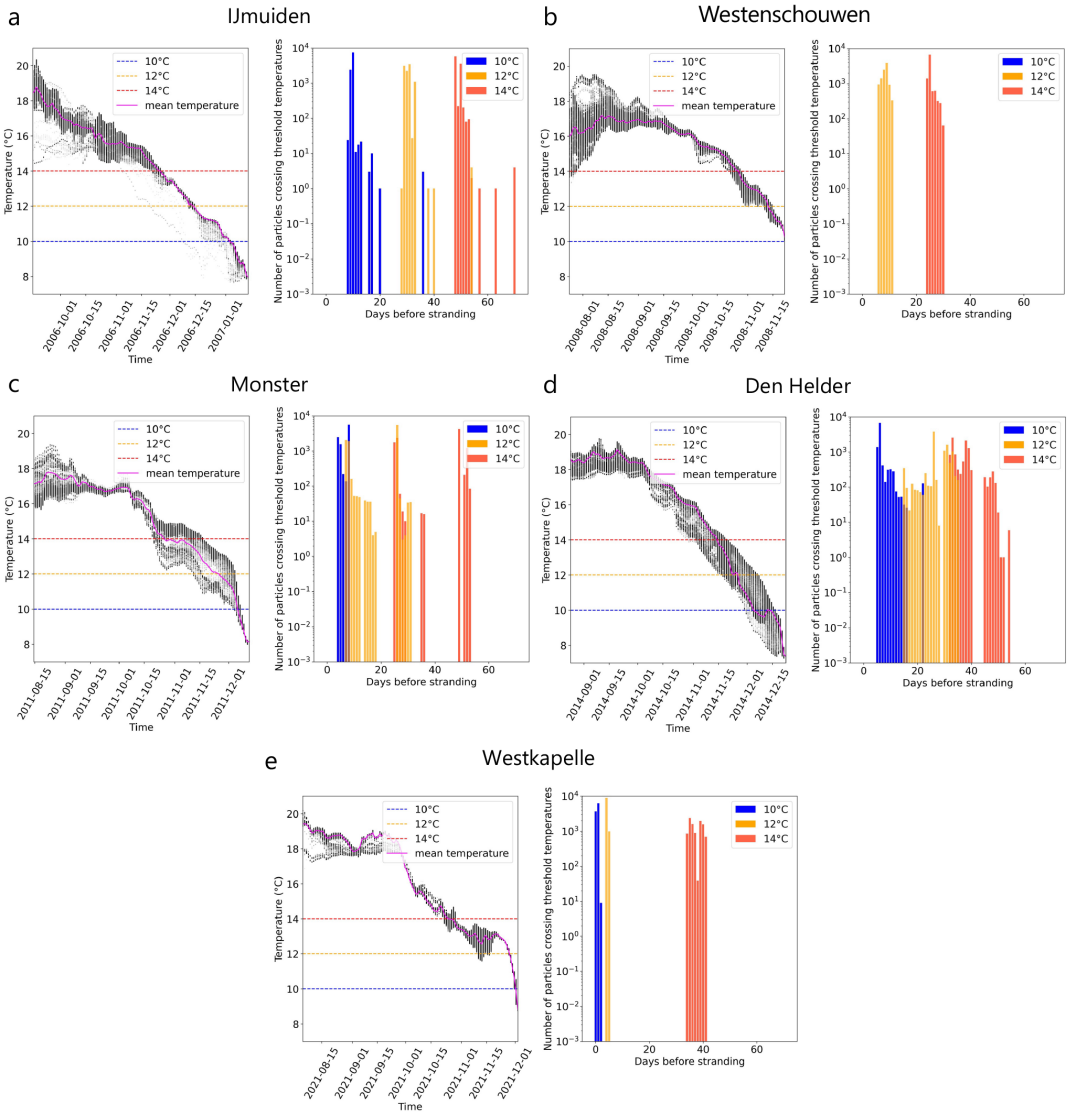
Days before stranding when threshold temperatures were crossed. For five recent Kemp’s ridley strandings in the Netherlands between 2007 and 2022 ( **A**– **E**), *(left)* the temperature distribution of all drifting particles *(black dots)* and temperature mean *(pink line)* over time before arriving at the stranding location using Lagrangian simulations with currents and Stokes drift only (i.e., no windage) are shown. Critical threshold temperatures analysed in this study ( *T _c_* : 10°, 12° and 14° C) are marked for reference *(dashed line)*. *(right)* Number of particles crossing threshold temperatures for the last time before stranding.

In the simulations due to currents and Stokes drift only (i.e., with a windage of 0.0%), particle trajectories always drop below threshold temperatures ( *T _c_*) of 10°, 12°and 14°C within the southern North Sea region ( Figure 2) except the stranding at IJmuiden, where particles experienced temperatures below 14°C even before crossing the English Channel ( Figure 2a). As expected, the distance from the stranding location decreases as the lower thresholds are crossed ( Figure 2). The continuous - and in the case of the strandings at Monster and Westkapelle steep - decrease in ocean surface temperature along the trajectories as particles approach the stranding location can also be observed in the temperature time series ( Figure 3a–e *(left)*).

In most cases, stranding occurred once the *T _c_* = 10°C threshold was crossed by particles. For IJmuiden, Monster, and Den Helder, temperatures of approximately 8°C were experienced by particles a few days before strandings ( Figure 3a,c,d). However, for the stranding at Westenschouwen, the ocean surface temperature along the trajectories never dropped below *T _c_* = 10°C ( Figure 3b *(left)*). Most particles crossed the lowest *T _c_* = 10°C a few days (Monster and Westkapelle) to almost two weeks (IJmuiden and Den Helder) before stranding ( Figure 3a,c–e *(right)*). Particles crossed *T _c_* = 12°C approximately a few days to a month before stranding. Finally, particles crossed *T _c_* = 14°C three to six weeks before stranding.

### Simulations with windage

**Figure 4.**
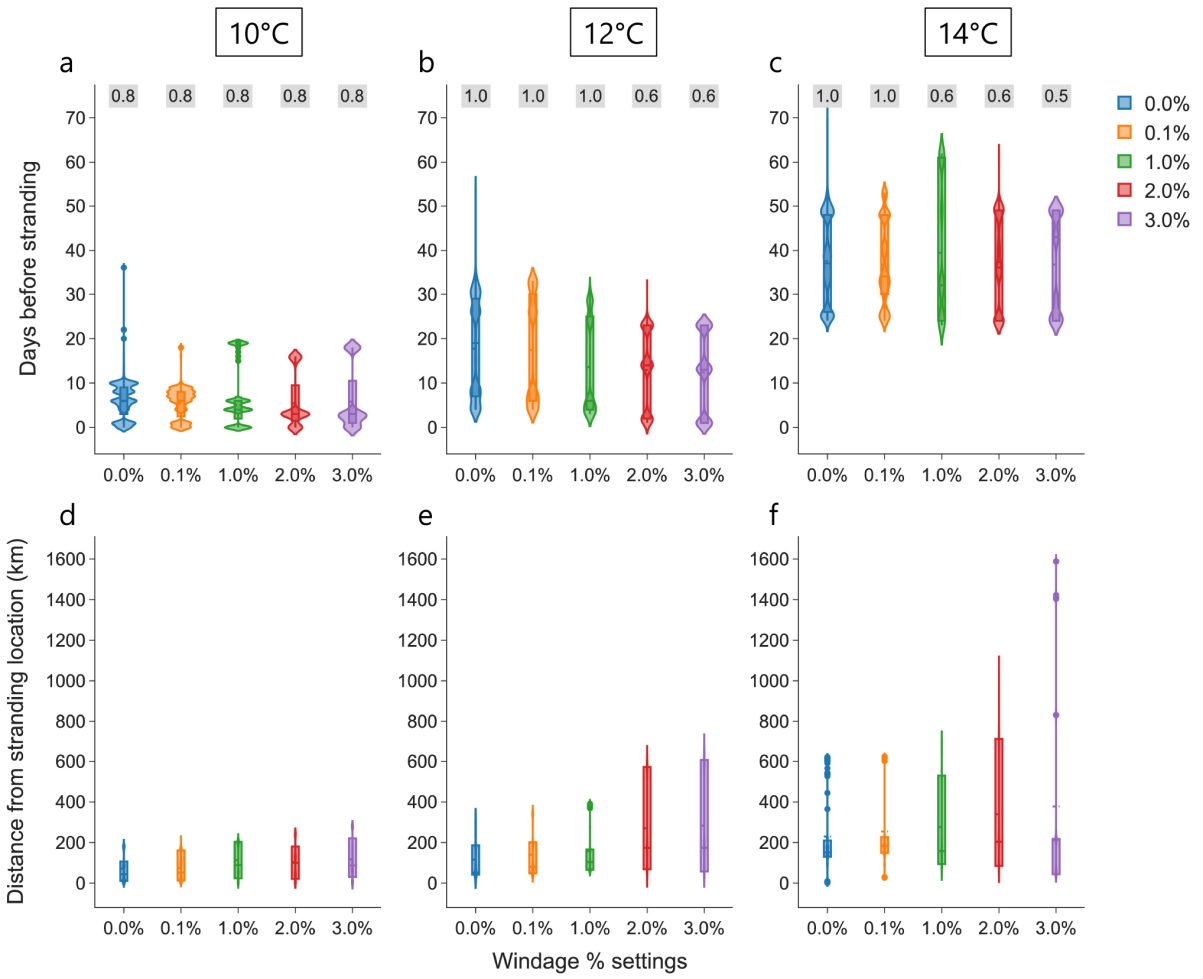
Effect of windage settings. Violin plots of days before stranding ( **a**– **c**) and distance of each particle from the respective stranding locations ( **e**– **f**) when a threshold temperature is crossed under the influence of currents, Stokes drift and different windage settings (0.0%, 0.1%, 1.0%, 2.0%, and 3.0%) considered in the analysis is shown. The text in *grey* box on top represents the proportion of total number of particles ( *n* = 50,000) crossing the threshold at a windage setting.

Simulations from all the stranding locations are combined to investigate the additional effect of four different percentages of 10 m atmospheric wind on the transport of particles: 0.1%, 1.0%, 2.0%, and 3.0%. For each windage setting across all the strandings, the time and distance between cold-stunning and stranding increases with the *T _c_* ; a similar trend to the case without windage ( Figure 4). For the particles that cross the *T _c_* = 10°C threshold, the mean time between cold-stunning and stranding varies marginally with increasing windage and ranges between 5.4 and 6.9 days ( Figure 4a). In addition, the mean distance of the stranding location from the cold-stunning locations shows an increase from 71.4 to 117.1 km with increasing windage ( Figure 4d). In the Westenschouwen stranding, particles did not cross the *T _c_* = 10°C threshold for any windage setting (see Figure3b *(left)*), therefore they were excluded from the statistics in Figure 4a,d. At the higher *T _c_* = 12°C threshold, the time between cold-stunning and stranding shows a small decrease in the mean from 17.7 to 12.4 days for increasing windage ( Figure 4b), while it marginally varies for *T _c_* = 14°C ( Figure 4c), similar to *T _c_* = 10°C. The mean distance from the stranding location increased with windage from 115.8 to 283.1 km and from 229.7 to 377.5 km for *T _c_* = 12°C and 14°C, respectively, and was considerably for higher windage of 2.0% and 3.0% ( Figure 4e,f). Similar to Figure 4, a summary of the windage effect on cold-stunning events for each stranding location is provided in Supplementary Information ^60^.

Ideally, particles should cross the threshold temperatures faster as the windage increases due to stronger drift, but no clear signature is observed in Figure 4a-c. This can be explained by the variable effect of windage on cold-stunning events with different temperature thresholds even for the individual strandings ^60^. For example, a clear decrease in time is visible for the IJmuiden stranding from 9.8 to 2.3 days in case of *T _c_* = 10°C, while it is variable for *T _c_* = 12°C and 14°C. For the same stranding, we also observe that the distribution of particle distance did not increase linearly with increasing windage for different temperature thresholds. We speculate that this variability results from the differences in the hydrodynamics of the study region over different stranding years. Note that the fraction of particles that crossed a certain *T _c_* also decreases with high windage at higher *T _c_* (see Figure 4). This can be primarily attributed to the increasing number of beached particles in the complex coastal regions and islands in the simulations: ~2–4% and ~50% of *n* = 50, 000 particles beached at low and high windage, respectively. High windage (in addition to currents and Stokes drift) can transport particles further into the land which cannot be successfully displaced to the sea by the anti-beaching kernel. In the case of the strandings at Monster and Westkapelle with 2.0–3.0% windage, particles originating from the UK coast with low ocean temperatures arrived at the stranding locations within a few days, without encountering temperatures above 10°C (see the animations and analysis output in 60).

## 4 Discussion

In all the seven historical strandings on the Dutch coast until 2022, juvenile Kemp’s ridley turtles were found alive at the time of reporting. However, only two turtles could be successfully rehabilitated and released in the Gulf of Mexico, while the others didn’t survive for more than a few days ^10– 12, 39^. The initial condition of the turtle at the time of stranding, the first response by observers, and the time lag in arriving at a rehabilitation facility add to the complexity of implementing rehabilitation protocols, particularly in locations far from their native habitat, where such occurrences are rare ^10^. While the role of wind and waves is suggested for the drift of floating cold-stunned turtles ^23, 24, 26^, we are the first to use Lagrangian simulations to model their backward in time drift with the surface currents including the effect of tides, waves, and wind from the stranding location along with temperature sampling to study the five recent Kemp’s ridley strandings in the Netherlands. Although we focus only on Kemp’s ridleys, we encourage the use of this method for strandings of other turtle species and/or regions, considering their species- and age-specific thermal constraints ^22^.

We show that turtles might have continuously encountered ocean surface temperatures below 14°C from locations most likely in the southern North Sea and up to the English Channel ( Figure 2). As previously noted in 10, these simulations further support the notion of Kemp’s ridley juvenile turtles reaching the North Sea via the English Channel. For the stranding at Westenschouwen in November 2008, it was observed that the juvenile Kemp’s ridley turtle might not have faced temperatures below 10°C indicating that temperatures between 10–12°C can also result in stranding. Differences in physiological conditions can also be responsible for stranding in this case but its analysis is beyond the scope of this study.

The decreasing distance from the stranding location and temperature as particles approached the stranding location ( Figure 2 and Figure 3) indicates that if the turtles start developing symptoms of cold stunning at 14°C, they are likely to face even lower temperatures, which could deteriorate their condition even more. Hence, turtles are unable to escape from the southern North Sea region to the relatively warmer Atlantic waters ( Figure 1). For each threshold temperature and all the strandings, increasing windage values resulted in an increase in distance; however, it did not have much effect on the time between cold-stunning and stranding ( Figure 4). Furthermore, compared to the suggested windage range based on literature (0.3-1.2%) ^55, 56, 59^, high windage scenarios of 2 and 3% in addition to Stokes drift likely overestimate the drift velocities resulting in the beaching of a large number of simulated particles in some stranding scenarios.

The timescale between crossing the threshold temperatures and the stranding on the Dutch coast varied in different stranding events from (i) a day to three weeks for *T _c_* = 10°C, (ii) a few days to a month for *T _c_* = 12°C, and (iii) three weeks to two months for *T _c_* = 14°C. We note that minimum temperatures of approximately 8°C were sampled in the simulations, as also indicated in the mean temperature field shown in Figure 1. Hence, lethal temperatures (5-6.5°C) ^22^ were not encountered by particles across all the windage scenarios, and low metabolism in cold temperatures ^18^ might explain how these turtles survived this transport over several days. However, it is unclear how long juvenile turtles can endure these conditions in the natural environment.

The possible timescales of turtles drifting in a cold-stunned state for a few days to two to three weeks (below 10°C) might indicate similar physiological conditions to those in multiple strandings reported within a day to up to a few weeks of temperatures between 8° and 11°C in the east US coast, e.g. as observed in 24 and 25. This suggests similar protocol requirements for the rehabilitation of Kemp’s ridley turtles in the Netherlands. Therefore, it is recommended that rehabilitation protocols are adequately implemented and individuals are further tracked upon release to assess the long-term impact of rehabilitation ^32, 33, 62^. For future strandings, the approach used in this study can even be applied to obtain real-time insights on the individual’s history in the cold-stunned state and accordingly take necessary steps for rehabilitation. Post-rehabilitation, the released turtles can be monitored for movement, feeding, diving, and interaction behaviour with other turtles, when equipped with satellite transmitters ^34, 62^. In addition, these rehabilitation opportunities should be used to raise awareness about threats faced by wild species and, educate and engage the local public towards collaborative conservation efforts ^33^.

Even though Kemp’s ridley juveniles are known to forage in the northeast Atlantic Ocean ^28, 62^, their transport into the North Sea is considered an occasional drift ^10^. Additional research could investigate the seasonal food availability in the southern North Sea to assess if these turtles were possibly feeding in this region before the onset of winter. This is particularly relevant for assessing changes in species distribution due to a warming climate ^63^ and is likely to impact cold-stunning-induced strandings ^29^. Furthermore, it remains to be investigated if conclusions drawn from this study also apply to juvenile Kemp’s ridley strandings on other western European coasts.

## Ethics and consent

Ethical approval and consent were not required.

## Data Availability

The Copernicus Marine Service ocean data used in this study can be downloaded from https://doi.org/10.48670/moi-00059 ^44^ and https://doi.org/10.48670/moi-00060 ^46^. The ERA 5 wind data can be downloaded from https://doi.org/10.24381/cds.adbb2d47 ^48^. Data publication platform of Utrecht University: kemps_ridleys_dutch_strandings. https://doi.org/10.24416/UU01-6NPX5Y ^60^. This project contains the following underlying data: File **simulation_output_files.zip**:Simulation outputs for different windage setting analysed in this study. Windage settings: 0.0%, 0.1%, 1.0%, 2.0% and 3.0% are abbreviated as 0pWind, 01pWind, 1pWind, 2pWind and 3pWind, respectively. Same structure is used in the folders below. Folder **animations/xpWind**: Animations of simulations are available in this folder within subfolders of each windage setting. Folders **analysis/xpWind**: Analysis of cold stunning event for each particle per simulation are available in subfolders for each windage setting. Folder **analysis/windage_effect_per_station**: Effect of each windage setting on the extracted cold stunning event is shown for each station. Data are available under the terms of the Creative Commons Attribution 4.0 International license (CC-BY 4.0). Analysis code available from: https://github.com/OceanParcels/Kempsridley_turtle_strandings Archived analysis code at time of publication: https://zenodo.org/doi/10.5281/zenodo.10450425 ^49^ License: Data are available under the terms of the Creative Commons Attribution 4.0 International license (CC-BY 4.0).
