# Supplementary material for: Modelling drift of cold-stunned Kemp's ridley turtles stranding on the Dutch coast
Source: Open Res Eur. 2024 Oct 25;4:41. Originally published 2024 Feb 29. [Version 3] doi: 10.12688/openreseurope.16913.3 (PMC11415756; doi:10.12688/openreseurope.16913.3)
Supplement: Peer review history

# Reviewer report: Reviewer response for version 3

**DOI:** 10.21956/openreseurope.20310.r45500

**Published:** 2024-11-04

**Authors:** Philippe Gaspar

**Affiliations:** 1 Mercator Ocean, Mercator Océan International:, Toulouse, France

The answer to my comments and the resulting revised version of the paper are OK. I thus approve the revised paper for indexing.

Is the study design appropriate and does the work have academic merit?

Yes

Is the work clearly and accurately presented and does it cite the current literature?

Partly

If applicable, is the statistical analysis and its interpretation appropriate?

I cannot comment. A qualified statistician is required.

Are all the source data underlying the results available to ensure full reproducibility?

Yes

Are the conclusions drawn adequately supported by the results?

Partly

Are sufficient details of methods and analysis provided to allow replication by others?

Yes

Reviewer Expertise:

Physical oceanography, numerical modeling,sea turtle spatial ecology

I confirm that I have read this submission and believe that I have an appropriate level of expertise to confirm that it is of an acceptable scientific standard.

# Reviewer report: Reviewer response for version 2

**DOI:** 10.21956/openreseurope.19879.r44020

**Published:** 2024-09-28

**Authors:** Philippe Gaspar

**Affiliations:** 1 Mercator Ocean, Mercator Océan International:, Toulouse, France

In this paper, the final part of the trajectories of 5 juvenile Kemp’s ridley turtles, found stranded on the Dutch coast, are simulated and analyzed using numerical Lagrangian back-tracking. Simulations are performed assuming that these turtles were cold-stunned and thus drifted passively at the ocean surface before getting stranded. The two first reviewers largely commented the implications of this paper for the conservation of Kemp’s ridleys and the rehabilitation process to apply to stranded cold-stunned individuals. I will rather comment on the technical aspects of the simulations.

**Drift modeling basis**

 Ensembles of likely turtle trajectories are simulated using the state-of-the-art Parcels software. Drift velocities used to generate turtle trajectories are obtained by summing the Eulerian surface current (including tides), the windage and the surface Stokes drift.  The surface current and Stokes drift are obtained from ocean and wave model outputs available from the Copernicus Marine Service. The windage is parameterized as a percentage of the 10-m wind speed (U10) retrieved from the ERA5 atmospheric circulations reanalysis.

**Main comment**

 Use of such a complete formulation of the drift velocity based on high-quality inputs is unusual in juvenile sea turtle drift studies and certainly a strong point of this paper.  

 My main comment concerns the range of windage estimates (0 to 3 % of U10) tested in the simulations presented here.

 These windage estimates  were selected following  Lebreton et al. (2018) [ref 3] and  Vogt-Vincent et al. (2023) [ref 8] who used these same estimates to test the sensitivity to windage of their marine debris dispersal simulations. Interestingly, Lebreton et al. [ref 3] concluded that the best simulations were obtained with a windage coefficient of 0 % while Vogt-Vincent did not conclude about the best windage estimate but noted that “there are limited publicly available estimates of appropriate windage coefficients for common classes of marine debris”.

 The scarcity of windage coefficient estimates is largely due to the fact that separate estimation of the Stokes drift and the windage is rather difficult as these two components of the drift velocity are highly correlated (Breivik et al., 2011) [ref 1]. Estimation of the leeway (the sum of the windage and the Stokes drift) is easier and thus more commonly found in the literature. Published estimates of the leeway of small to medium marine debris typically vary between 1 and 3 % of U10, up to 5 % for larger debris (Duhec et al., 2015;[ref 2] Maximenko et al., 2018 [ref 4]).  Similar leeway coefficients have been obtained for surface floating turtles. Nero et al. (2013) estimated the leeway of a passive (moribund) juvenile Kemp’s ridley turtle to be close to 3.5 % of U10  . Santos et al. (2018)[ref 7] estimated the leeway of larger floating sea turtle carcasses to be between 1.1 and 3.6 % of U10.

 This rapid literature overview suggests that 1 to 3 % of U10 is a reliable estimate of the leeway (for both medium marine debris and sea turtles) but not for the windage alone. Adding an estimated Stokes drift to this quantity inevitably leads to an overestimation of the leeway. This might very well be the reason why Lebreton et al. get better results using a zero windage coefficient and why the simulations of Manral et al., performed with windages above 1 % U10 yield unusually high percentages of beached particles, suggesting use of non-realistic drift velocities (on top of numerical issues linked to complex shorelines).

 The ultimate question is then: what is a reasonable estimate of the windage of juvenile, surface floating, sea turtles? Although this has never been directly measured in the field, the modeling work of Röhrs et al. (2012)[ref 6] on the leeway of small (diameter = 35 cm) spherical half-submerged surface buoys can provide some indication. It shows that, in such buoys, the windage accounts for about one third of the leeway.  A similar ratio probably holds for small sea turtles which size, shape and buoyancy are not very different from those of these buoys. Since turtle’s leeway estimates vary estimates between 1 and 3.6 % of U10, one can reasonably assume that their windage varies between 0.3 and 1.2 %.  Such a range of windage estimates is worth testing. Larger windages can still be tested but acknowledging they are most probably overestimates.

**Recommendation**

 I recommend that this paper be accepted, provided that estimation of the windage is more thoroughly discussed, following the comment above. This might also help the authors justify why they chose to mostly present results (Fig. 2 and 3) from simulations performed with no windage (they are probably more realistic than simulations with a 2 or 3 % windage on top of a modeled surface Stokes drift)

 Simulations with high (> 1 %) windage coefficients on top of the Stokes drift can still be presented but clearly mentioning that the drift velocity is most likely overestimated.

Minor comment:

 Page 8 (below Fig. 3): in “ *particles beached at low and high wind-age respectively*”, “ *age*” shall be replaced by “speed”.

**References**

 Breivik, Ø., et al 2011 [ref 1]

 Duhec, A.V., et al  2015 [ref 2]

 Lebreton, L., et al  2018 [ref 3]

 Maximenko, N., et al  2011 [ref 4]

 Nero, R., et al  2013 [ref 5]

 Röhrs, J., et al  2012 [ref 6]

 Santos, B.S., et al  2018 [ref 7]

 Vogt-Vincent., et al  2023 [ref 8]

Is the study design appropriate and does the work have academic merit?

Yes

Is the work clearly and accurately presented and does it cite the current literature?

Partly

If applicable, is the statistical analysis and its interpretation appropriate?

I cannot comment. A qualified statistician is required.

Are all the source data underlying the results available to ensure full reproducibility?

Yes

Are the conclusions drawn adequately supported by the results?

Partly

Are sufficient details of methods and analysis provided to allow replication by others?

Yes

Reviewer Expertise:

Physical oceanography, numerical modeling,sea turtle spatial ecology

I confirm that I have read this submission and believe that I have an appropriate level of expertise to confirm that it is of an acceptable scientific standard, however I have significant reservations, as outlined above.

## References

[1] : Wind-induced drift of objects at sea: The leeway field method. *Applied Ocean Research* .2011;33(2) : 10.1016/j.apor.2011.01.005 100-109 10.1016/j.apor.2011.01.005

[2] : Composition and potential origin of marine debris stranded in the Western Indian Ocean on remote Alphonse Island, Seychelles. *Mar Pollut Bull* .2015;96(1-2) : 10.1016/j.marpolbul.2015.05.042 76-86 10.1016/j.marpolbul.2015.05.042 26024564

[3] : Evidence that the Great Pacific Garbage Patch is rapidly accumulating plastic. *Sci Rep* .2018;8(1) : 10.1038/s41598-018-22939-w 4666 10.1038/s41598-018-22939-w 29568057 PMC5864935

[4] : Numerical simulations of debris drift from the Great Japan Tsunami of 2011 and their verification with observational reports. *Mar Pollut Bull* .2018;132: 10.1016/j.marpolbul.2018.03.056 5-25 10.1016/j.marpolbul.2018.03.056 29728262

[5] : Using an ocean model to predict likely drift tracks of sea turtle carcasses in the north central Gulf of Mexico. *Endangered Species Research* .2013;21(3) : 10.3354/esr00516 191-203 10.3354/esr00516

[6] : Observation-based evaluation of surface wave effects on currents and trajectory forecasts. *Ocean Dynamics* .2012;62(10-12) : 10.1007/s10236-012-0576-y 1519-1533 10.1007/s10236-012-0576-y

[7] : Consequences of drift and carcass decomposition for estimating sea turtle mortality hotspots. *Ecological Indicators* .2018;84: 10.1016/j.ecolind.2017.08.064 319-336 10.1016/j.ecolind.2017.08.064

[8] : Sources of marine debris for Seychelles and other remote islands in the western Indian Ocean. *Mar Pollut Bull* .2023;187: 10.1016/j.marpolbul.2022.114497 114497 10.1016/j.marpolbul.2022.114497 36646000

# Response

**Published:** 2024-10-16

**Authors:** Darshika Manral

**Affiliations:** Utrecht University, Utrecht, Utrecht, The Netherlands

Dear Reviewer,  

 We thank you for your detailed feedback on the manuscript. We have adjusted the text in the manuscript based on your feedback. Following sentences were added (and adjusted) to the Methods section: Particles are advected with a combined flow obtained from summing ocean currents (with tides), Stokes drift, and windage as a fraction (i.e., windage factor) of the 10 m atmospheric wind, similar to the formulation used for modeling drift of floating marine debris in 51 and 52. The combination of Stokes drift and windage combined defines the ‘leeway’ drift of a floating object relative to the ambient current ^57, 58^. However, since wave models that compute Stokes drift are not always available, and Stokes drift is indirectly driven by local wind, just like windage, the resulting leeway is for simplicity often estimated from the 10 m wind ^59, 58^. Estimates of leeway for floating sea turtle carcasses range from 1 to 3.6% of 10 m wind ^60, 61^, similar to that of medium-sized marine debris ^62, 63^. Furthermore, in an experiment and modeling based study by 64, it was shown that windage accounts for about one-third of the leeway for a half submerged spherical buoy (diameter = 35 cm), which is comparable to the length of juvenile Kemp’s ridley turtles. This suggests that windage contribution likely varies between 0.3 and 1.2% for these turtles. Given the uncertainties of leeway contribution to the drift of the alive juvenile turtles, we perform a sensitivity analysis with five settings of windage factor: 0.0% (i.e., no windage), 0.1%, 1.0%, 2.0%, and 3.0%.   In the Discussion, we added the following sentence: Furthermore, compared to the suggested windage range based on literature (0.3-1.2%) ^60, 61, 64^, high windage scenarios of 2 and 3% in addition to Stokes drift likely overestimate the drift velocities resulting in the beaching of a large number of simulated particles in some stranding scenarios.   As for the minor comment, it is possible that the confusion arose because of the hyphen in ‘wind-age’. Therefore, no action was taken as use of term windage is quite consistent throughout.   We look forward to your further comments.  

 Best regards, Darshika Manral and co-authors

# Reviewer report: Reviewer response for version 2

**DOI:** 10.21956/openreseurope.19879.r42948

**Published:** 2024-09-20

**Authors:** Lesley Stokes

**Affiliations:** 1 Southeast Fisheries Science Center,, NOAA/National Marine Fisheries Service,, Miami,, Florida,, USA

The authors have addressed all relevant feedback, and the manuscript is ready for acceptance.

Is the study design appropriate and does the work have academic merit?

Yes

Is the work clearly and accurately presented and does it cite the current literature?

Yes

If applicable, is the statistical analysis and its interpretation appropriate?

I cannot comment. A qualified statistician is required.

Are all the source data underlying the results available to ensure full reproducibility?

Yes

Are the conclusions drawn adequately supported by the results?

Yes

Are sufficient details of methods and analysis provided to allow replication by others?

Yes

Reviewer Expertise:

sea turtle biology

I confirm that I have read this submission and believe that I have an appropriate level of expertise to confirm that it is of an acceptable scientific standard.

# Reviewer report: Reviewer response for version 2

**DOI:** 10.21956/openreseurope.19879.r42949

**Published:** 2024-08-27

**Authors:** Casper Van de Geer

**Affiliations:** 1 Centre for Ecology and Conservation, University of Exeter (Ringgold ID: 151752), Penryn, Cornwall, UK

The authors have addressed feedback to where the paper is ready for acceptance.

Is the study design appropriate and does the work have academic merit?

Yes

Is the work clearly and accurately presented and does it cite the current literature?

Partly

If applicable, is the statistical analysis and its interpretation appropriate?

I cannot comment. A qualified statistician is required.

Are all the source data underlying the results available to ensure full reproducibility?

Yes

Are the conclusions drawn adequately supported by the results?

Yes

Are sufficient details of methods and analysis provided to allow replication by others?

Partly

Reviewer Expertise:

Sea turtle ecology and conservation

I confirm that I have read this submission and believe that I have an appropriate level of expertise to confirm that it is of an acceptable scientific standard.

# Reviewer report: Reviewer response for version 1

**DOI:** 10.21956/openreseurope.18278.r41256

**Published:** 2024-06-28

**Authors:** Casper Van de Geer

**Affiliations:** 1 Centre for Ecology and Conservation, University of Exeter (Ringgold ID: 151752), Penryn, Cornwall, UK

**Overall:**

 An interesting paper that investigates strandings of a critically endangered species of sea turtle, Kemp’s ridley (Lk), a long way from their usual range.

 It would be good to know why the authors chose to only look at Kemp’s ridley turtles (Lk), when there are also strandings data from other species, e.g., loggerhead turtles (as seen on waarneming.nl).

 Certain parts of the paper could use some copyediting.

**Abstract:**

 Consider editing the Background section of the Abstract to (1) remove repetition that there are Lk strandings on the Dutch coast and (2) clarify how your research could help improve the rehabilitation process.

**Introduction:**

 Suggest indicating the year of the population estimate cited in text.

**Methods:**

 Were the data from waarneming.nl or from literature sources 10-12 and 35? Are the strandings reported in these literature sources captured in the waarneming.nl database? If so, then a brief recognition of the usefulness of waarneming.nl will suffice and this paragraph can be made clearer and shorter.

 CCL stands for “curved carapace length”, please correct the mistake in the definition. Also please specify what type of CCL was taken. If this is unknown, please note that in the text.

 For reference, please see: Bolten AB (1999) 

 Please report resolutions of data that were used in both degrees and kilometers, for ease of interpretation.

 Section “Defining cold stunning events” contains information that should be, or already is, in the introduction. This section can be made shorter and merged with the previous methods section.

 Python package “cartopy” is used, so it should be added to the references. The same goes for other analysis software that was used.

 I am not familiar with the technicalities of Lagrangian modelling and as such cannot comment on whether the methods used are appropriate.

**Results:**

 In the discussion, the authors note that the magnitude of windage did “not have much effect on the time between cold-stunning and stranding.” Can a statistical test be done to investigate where the magnitude becomes a significant factor?

**Discussion:**

 Please check statements are underpinned with literature. For instance, the last sentence of the second paragraph should be supported by findings from other studies.

 The authors mention that individuals should be monitored post-release to assess survival rates and possible contribution to the Lk population, which is a point well-made. However, this is not further elaborated in the paper.

 Comparison to other similar studies would be beneficial, especially to compare methods. For instance: Hart et al. (2006)

**Figures:**

 Figure 1: suggest making the place markers (red dots) more clear. Perhaps change to something more colour-blind friendly (I am colour blind) or add black outline to the dots.

 Also suggest adding reference to the animated particles in the legend.

Is the study design appropriate and does the work have academic merit?

Yes

Is the work clearly and accurately presented and does it cite the current literature?

Partly

If applicable, is the statistical analysis and its interpretation appropriate?

I cannot comment. A qualified statistician is required.

Are all the source data underlying the results available to ensure full reproducibility?

Yes

Are the conclusions drawn adequately supported by the results?

Yes

Are sufficient details of methods and analysis provided to allow replication by others?

Partly

Reviewer Expertise:

Sea turtle ecology and conservation

I confirm that I have read this submission and believe that I have an appropriate level of expertise to confirm that it is of an acceptable scientific standard, however I have significant reservations, as outlined above.

## References

[1] : Interpreting the spatio-temporal patterns of sea turtle strandings: Going with the flow. *Biological Conservation* .2006;129(2) : 10.1016/j.biocon.2005.10.047 283-290 10.1016/j.biocon.2005.10.047

[2] : Techniques for measuring sea turtles. In: Research and Management Techniques for the Conservation of Sea Turtles. *IUCN/SSC Marine Turtle Specialist Group Publication* .1999;110-114

# Response

**Published:** 2024-07-02

**Authors:** Darshika Manral

**Affiliations:** Utrecht University, Utrecht, Utrecht, The Netherlands

Thank you for your review comments. I am on a research cruise currently and will take at least 3-4 weeks to respond with the next version.

# Response

**Published:** 2024-08-05

**Authors:** Darshika Manral

**Affiliations:** Utrecht University, Utrecht, Utrecht, The Netherlands

Dear Reviewer, We thank you for your feedbacks on the manuscript. They were of great help in improving the manuscript. We have submitted the updated manuscript along with responses to each comment given below. Please note some additonal changes that have been made:

- Throughout: We clarify that we are investigating Kemp’s rdidley strandings only until 2022.
- In the conclusion: We have added a statement to emphasize on the need for understanding changes to species distribution.
  “Additional research could investigate the seasonal food availability in the southern North Sea to assess if these turtles were possibly feeding in this region prior to the onset of winter . This is particularly relevant for assessing changes in the species distribution with a warming climate ^52^ and is likely to imapct cold-stunning induced strandings ^29^ .”
- Grammar corrections and some sentences have been reformed for clarity

We look forward to your further comments. Best regards, Darshika Manral and co-authors **Overall:**

 An interesting paper that investigates strandings of a critically endangered species of sea turtle, Kemp’s ridley (Lk), a long way from their usual range.

 It would be good to know why the authors chose to only look at Kemp’s ridley turtles (Lk), when there are also strandings data from other species, e.g., loggerhead turtles (as seen on waarneming.nl).

   Certain parts of the paper could use some copyediting.

**Response:**We have addressed the point on choice of turtle species in the introduction (last paragraph): “The strandings of juvenile Kemp’s ridley turtles in the Netherlands are unique, though rare, as they always stranded alive, unlike loggerhead, green, and leatherback turtles, which arrive at different life stages and are often dead or in various stages of decomposition ^10^. This higher survival rate may be attributed to the observed higher tolerance of Kemp’s ridleys to lethal temperatures of 5-6.5°C for 20-24 hours compared to loggerhead or green turtles ^22^. Secondly, Kemp’s ridleys stranding in the Netherlands belong to a single population originating from the Gulf of Mexico ^2^, while species such as loggerhead turtles, which have multiple subpopulations in the Atlantic Ocean ^54^, can arrive in the Netherlands from different regions. This conclusion is based on an analysis of loggerhead turtles that stranded in the Netherlands in 2023 and 2024, conducted by the Gendika B.V. laboratory in Veendam (Netherlands), using the method described in 56 (unpublished).”

**Abstract:**

 Consider editing the Background section of the Abstract to (1) remove repetition that there are Lk strandings on the Dutch coast and (2) clarify how your research could help improve the rehabilitation process.

**Response: **We have updated the abstract where we removed some of the repetitions. For the second point we added the following sentence: “The estimate of cold-stunned drift duration of the turtles provides additional knowledge about their health status at the time of stranding.” **Introduction:**

 Suggest indicating the year of the population estimate cited in text.

**Response: **We have updated the text: “With 22,341 mature individuals of a single known population and declining numbers over the past three generations, they are critically endangered according to the latest International Union for Conservation of Nature (IUCN) assessment in 2019 ^2^ .”

**Methods:**

 Were the data from waarneming.nl or from literature sources 10-12 and 35? Are the strandings reported in these literature sources captured in the waarneming.nl database? If so, then a brief recognition of the usefulness of waarneming.nl will suffice and this paragraph can be made clearer and shorter.

**Response: **Some clarifications have been made to the sources and the paragraph has been edited:   “Seven strandings of Kemp’s ridley turtles have been reported in the Netherlands until 2022 ^11^^, ^^12^^, ^^35^ . Two of those strandings took place in December of 1954 and 1970, while the others happened in this century ( Table 1). The 2007 stranding location was obtained from 53 and the rest from waarneming.nl, a citizen-science-based online platform where different flora and fauna observations in the Netherlands are curated. These strandings were also confirmed in the literature, with details about turtles’ status ^10^^– ^^12^. All the stranded turtles were alive at the time of reporting, which might reflect the accessibility of flat and sandy Dutch beaches from nearby population centers, increasing the likelihood that these turtles were not stranded for long before being found.”

 CCL stands for “curved carapace length”, please correct the mistake in the definition. Also please specify what type of CCL was taken. If this is unknown, please note that in the text.

 For reference, please see: Bolten AB (1999) 

**Response: **The text has been updated to: The curved carapace length notch to tip (CCLn-t) (see Figure 1b in 55) of these individuals was between 20–30 cm ^10^^– ^^12^^, ^^35^ and were identified as juveniles ^36^ .  

 Please report resolutions of data that were used in both degrees and kilometers, for ease of interpretation.

**Response: **The text has been updated: “Daily mean ocean surface currents that include the effect of tides are obtained from the reanalysis data for the European North West Shelf along with ocean surface temperatures, all available since 1993 at the spatial resolution of 0.111° × 0.067° (~ 7 km) ^38^ . In addition, the effect of Stokes drift (a net transport in the direction of wave propagation due to surface waves) ^39^ on the transport of these particles is accounted for using three-hourly data from 40, available since 1980 at ~1.5 km resolution (0.0135° × 0.0303°). Since floating turtles might be partially exposed to the atmospheric wind, the effect of wind on their transport is also investigated ^41^ . Three-hourly wind at 10 m above sea level is obtained from ERA5 reanalysis data at 0.25° × 0.25° resolution (~17.51 km × 27.83 km, computed at 51° N) ^42^ .” Section “Defining cold stunning events” contains information that should be, or already is, in the introduction. This section can be made shorter and merged with the previous methods section.

**Response: **The repetitive part has been removed. However, we would like to keep the remaining text in a separate subsection as it highlights a key aspect of our analysis: selected temperature thresholds. The updated paragraph is as follows: “ **Extracting the cold-stunning event** Due to the floating behaviour of the cold-stunned Kemp’s ridley turtles, their movement is simulated as passively drifting virtual particles at the ocean’s surface (also suggested in 23 and 26). The most recent event before stranding when the interpolated ocean surface temperature on a particle trajectory drops below a threshold temperature is extracted from its trajectory and marked as a cold-stunning event, similar to the approach used in 26. We assume that turtles can swim above the threshold temperature and hence, their movement cannot be simulated with passive drift. In nature, this switch from active to passive drift is indeed gradual and depends on the health status of the individual turtle; therefore, we examine three threshold temperatures ( T _c_ : 10°C, 12°C and 14°C) in this analysis. Our approach differs from other studies where a single temperature (10.5°C) was selected to consider turtles as cold-stunned ^26^, or where it was assumed that young turtles in the first year of their drift die if the temperature drops below 10°C ^51^.“   Python package “cartopy” is used, so it should be added to the references. The same goes for other analysis software that was used. **Response: **cartopy citation is now added at #49 and is referenced in Figure 1 and 2. The updated text is as follows: “The land mask is obtained from the ocean model ^38^ and coastlines from python package cartopy ^49^. “ Reference for Parcels framework is already available at #37.

 I am not familiar with the technicalities of Lagrangian modelling and as such cannot comment on whether the methods used are appropriate.

**Results:**

 In the discussion, the authors note that the magnitude of windage did “not have much effect on the time between cold-stunning and stranding.” Can a statistical test be done to investigate where the magnitude becomes a significant factor?

**Response: **The selected range of windage used in our study is more practical for our use-case here, therefore, we don’t think we should test for higher windages. Higher percentages of 4-5% are used for buoys or robust empty bottles like objects (reference-45,46 in the manuscript). Instead, we have improved the description of the results elaborating on the wingage effects: “Simulations from all the stranding locations are combined to investigate the additional effect of four different percentages of 10 m atmospheric wind on the transport of particles: 0.1%, 1.0%, 2.0%, and 3.0%. For each windage setting across all the strandings, the time and distance between cold-stunning and stranding increases with the T _c_ ; a similar trend to the case without windage ( Figure 4). For the particles that cross the T _c_ = 10°C threshold, the mean time between cold-stunning and stranding varies marginally with increasing windage and ranges between 5.4 and 6.9 days ( Figure 4a). In addition, the mean distance of the stranding location from the cold-stunning locations shows an increase from 71.4 to 117.1 km with increasing windage ( Figure 4d). In the Westenschouwen stranding, particles did not cross the T _c_ = 10°C threshold for any windage setting (see Figure3b (left) ), therefore they were excluded from the statistics in Figure 4a,d. At the higher T _c_ = 12°C threshold, the time between cold-stunning and stranding shows a small decrease in the mean from 17.7 to 12.4 days for increasing windage ( Figure 4b), while it marginally varies for T _c_ = 14°C ( Figure 4c), similar to T _c_ = 10°C. The mean distance from the stranding location increased with windage from 115.8 to 283.1 km and from 229.7 to 377.5 km for T _c_ = 12°C  and 14°C, respectively, and was considerably for higher windage of 2.0% and 3.0% ( Figure 4e,f). Similar to Figure 4, a summary of the windage effect on cold-stunning events for each stranding location is provided in Supplementary Information ^47^.   Ideally, particles should cross the threshold temperatures faster as the windage increases due to stronger drift, but no clear signature is observed in Figure 4a-c. This can be explained by the variable effect of windage on cold-stunning events with different temperature thresholds even for the individual strandings ^47^. For example, a clear decrease in time is visible for the IJmuiden stranding from 9.8 to 2.3 days in case of T _c_ = 10°C, while it is variable for T _c_ = 12°C and 14°C. For the same stranding, we also observe that the distribution of particle distance did not increase linearly with increasing windage for different temperature thresholds. We speculate that this variability results from the differences in the hydrodynamics of the study region over different stranding years.

**Discussion:**

 Please check statements are underpinned with literature. For instance, the last sentence of the second paragraph should be supported by findings from other studies.

**Response: **Reference(#18) has been added.

 The authors mention that individuals should be monitored post-release to assess survival rates and possible contribution to the Lk population, which is a point well-made. However, this is not further elaborated in the paper.

**Response: **The following text has been added: “Post-rehabilitation, the released turtles can be monitored for movement, feeding, diving, and interaction behaviour with other turtles, when equipped with satellite transmitters ^34^^, ^^48^.”

 Comparison to other similar studies would be beneficial, especially to compare methods. For instance: Hart et al. (2006)

**Response: **We have compared our method to other studies including Hart et al. (2006) (references- 50, 26, 51) in the introduction: “Previously, pathways of cold-stunned or dead turtles have been studied using different methods like tracking of drift bottles experiment and satellite-tracked drifters released offshore, and forward and backward in time virtual particle simulations using ocean data ^50, 26,51^. In addition, these studies then analyse environmental factors like temperature, wind, and currents data to interpret the stranding patterns of cold-stunned turtles. Here, we combine these approaches to model the drift of cold-stunned turtles from their observed stranding locations and dates using surface currents, waves, and wind data, along with temperature sampling along the trajectory of virtual particles in a previously unstudied region.”

**Figures:**

 Figure 1: suggest making the place markers (red dots) more clear. Perhaps change to something more colour-blind friendly (I am colour blind) or add black outline to the dots. **Response: **Updated the Figure with black edges to the markers. Also suggest adding reference to the animated particles in the legend.

**Response: **We have added the following sentence to the caption of Figure 2: “The animations of the back-tracked particles are provided in 47.”

# Reviewer report: Reviewer response for version 1

**DOI:** 10.21956/openreseurope.18278.r39323

**Published:** 2024-05-28

**Authors:** Lesley Stokes

**Affiliations:** 1 Southeast Fisheries Science Center,, NOAA/National Marine Fisheries Service,, Miami,, Florida,, USA

Introduction: One comment is in reference to the line:  “In order to enhance the survival prospects of endangered and threatened species like Kemp’s ridley sea turtles, it is crucial to have every individual, particularly those nearing reproductive age, released back into the wild to maximize the potential for species preservation.” We don’t have evidence that rehabilitation of a relatively few number of stranded individuals will make a difference at a population level, so this statement may be controversial. I suggest a less definitive tone, stating that releasing individuals, particularly those nearing reproductive age, may be beneficial for species preservation, but not going so far as to suggest that it is crucial to have every individual rehabilitated and released.  The last line in the paragraph addressing sea turtle welfare and outreach is a much stronger supporting argument for the expense and effort of rehabilitation.

 Discussion: It may not be appropriate to say “However, only two… raising questions about the shortcomings in rehabilitation protocols or their adequate implementation.” There are many factors that could influence whether a turtle was successfully rehabilitated and transported back to the native habitat (a major undertaking).  The condition of the turtle at stranding is the most critical component, as the best rehabilitative care cannot overcome some cases where the turtle was in very poor condition at intake.  No information was given about the reasons that the other 5 turtles were not successfully released. Did they die in rehabilitation? Were they kept as unreleasable permanent residents due to logistical constraints preventing release back to the Gulf of Mexico? Was there not a facility with the resources or knowledge to properly care for the turtles?  A bit of context would make this a stronger section if you want to bring up shortcomings in the protocols, even just to introduce these factors influencing rehabilitation success and note that further description is beyond the scope of this manuscript.

Is the study design appropriate and does the work have academic merit?

Yes

Is the work clearly and accurately presented and does it cite the current literature?

Yes

If applicable, is the statistical analysis and its interpretation appropriate?

I cannot comment. A qualified statistician is required.

Are all the source data underlying the results available to ensure full reproducibility?

Yes

Are the conclusions drawn adequately supported by the results?

Yes

Are sufficient details of methods and analysis provided to allow replication by others?

Yes

Reviewer Expertise:

sea turtle biology

I confirm that I have read this submission and believe that I have an appropriate level of expertise to confirm that it is of an acceptable scientific standard, however I have significant reservations, as outlined above.

# Response

**Published:** 2024-07-02

**Authors:** Darshika Manral

**Affiliations:** Utrecht University, Utrecht, Utrecht, The Netherlands

Thank you for your review remarks. We will incorporate the suggested changes in the next version.

# Response

**Published:** 2024-08-05

**Authors:** Darshika Manral

**Affiliations:** Utrecht University, Utrecht, Utrecht, The Netherlands

Dear Reviewer, We thank you for your feedbacks on the manuscript. They were of great help in improving the manuscript. We have submitted the updated version along with responses to each comment given below. Please note some additonal changes that have been made:

- Throughout: We clarify that we are investigating Kemp’s rdidley strandings only until 2022.
- In the conclusion: We have added a statement to emphasize on the need for understanding changes to species distribution.
  “Additional research could investigate the seasonal food availability in the southern North Sea to assess if these turtles were possibly feeding in this region prior to the onset of winter *. This is particularly relevant for assessing changes in the species distribution with a warming climate ^52^ and is likely to imapct cold-stunning induced strandings ^29^ .”*
- Grammar corrections and some sentences have been reformed for clarity

We look forward to your further comments. Best regards, Darshika Manral and co-authors **Comment 1 ** Introduction: One comment is in reference to the line: *“In order to enhance the survival prospects of endangered and threatened species like Kemp’s ridley sea turtles, it is crucial to have every individual, particularly those nearing reproductive age, released back into the wild to maximize the potential for species preservation.” * We don’t have evidence that rehabilitation of a relatively few number of stranded individuals will make a difference at a population level, so this statement may be controversial. I suggest a less definitive tone, stating that releasing individuals, particularly those nearing reproductive age, may be beneficial for species preservation, but not going so far as to suggest that it is crucial to have every individual rehabilitated and released.  The last line in the paragraph addressing sea turtle welfare and outreach is a much stronger supporting argument for the expense and effort of rehabilitation.

**Response: **We have updated the text to the following: “Rehabilitating and releasing endangered or threatened species like Kemp’s ridley sea turtles into the wild may enhance their survival prospects and contribute to species conservation ^34^ , especially in the case of individuals nearing reproductive age."  

**Comment 2** Discussion:  It may not be appropriate to say “However, only two… raising questions about the shortcomings in rehabilitation protocols or their adequate implementation.” There are many factors that could influence whether a turtle was successfully rehabilitated and transported back to the native habitat (a major undertaking).  The condition of the turtle at stranding is the most critical component, as the best rehabilitative care cannot overcome some cases where the turtle was in very poor condition at intake.  No information was given about the reasons that the other 5 turtles were not successfully released. Did they die in rehabilitation? Were they kept as unreleasable permanent residents due to logistical constraints preventing release back to the Gulf of Mexico? Was there not a facility with the resources or knowledge to properly care for the turtles?  A bit of context would make this a stronger section if you want to bring up shortcomings in the protocols, even just to introduce these factors influencing rehabilitation success and note that further description is beyond the scope of this manuscript. **Response: **We have updated the text to the following: “However, only two turtles could be successfully rehabilitated and released in the Gulf of Mexico, while the others didn’t survive for more than a few days ^10^^-^^12^^, ^^35^. The initial condition of the turtle at the time of stranding, the first response by observers, and the time lag in arriving at a rehabilitation facility add to the complexity of implementing rehabilitation protocols, particularly in locations far from their native habitat, where such occurrences are rare ^10^.”
